# The Weak Complex between RhoGAP Protein ARHGAP22 and Signal Regulatory Protein 14-3-3 Has 1∶2 Stoichiometry and a Single Peptide Binding Mode

**DOI:** 10.1371/journal.pone.0041731

**Published:** 2012-08-28

**Authors:** Shu-Hong Hu, Andrew E. Whitten, Gordon J. King, Alun Jones, Alexander F. Rowland, David E. James, Jennifer L. Martin

**Affiliations:** 1 Institute for Molecular Bioscience, Division of Chemistry and Structural Biology, The University of Queensland, Brisbane, Queensland, Australia; 2 Garvan Institute of Medical Research, Darlinghurst, Sydney, New South Wales, Australia; Griffith University, Australia

## Abstract

ARHGAP22 is a RhoGAP protein comprising an N-terminal PH domain, a RhoGAP domain and a C-terminal coiled-coil domain. It has recently been identified as an Akt substrate that binds 14-3-3 proteins in response to treatment with growth factors involved in cell migration. We used a range of biophysical techniques to investigate the weak interaction between 14-3-3 and a truncated form of ARHGAP22 lacking the coiled-coil domain. This weak interaction could be stabilized by chemical cross-linking which we used to show that: a monomer of ARHGAP22 binds a dimer of 14-3-3; the ARHGAP22 PH domain is required for the 14-3-3 interaction; the RhoGAP domain is unlikely to participate in the interaction; Ser16 is the more important of two predicted 14-3-3 binding sites; and, phosphorylation of Ser16 may not be necessary for 14-3-3 interaction under the conditions we used. Small angle X-ray scattering and cross-link information were used to generate solution structures of the isolated proteins and of the cross-linked ARHGAP22:14-3-3 complex, showing that no major rearrangement occurs in either protein upon binding, and supporting a role for the PH domain and N-terminal peptide of ARHGAP22 in the 14-3-3 interaction. Small-angle X-ray scattering measurements of mixtures of ARHGAP22 and 14-3-3 were used to establish that the affinity of the interaction is ∼30 µM.

## Introduction

The Rho (Ras homologous) GTPases (or G-proteins) belong to the Ras superfamily of small GTP-binding proteins that switch between inactive GDP-bound and active GTP-bound forms [1], [2]. Three members of the Rho family of GTPases, RhoA, Rac1 and CDC42 are well characterized and function in diverse cellular processes, including cytoskeletal organization, gene transcription, secretion, and endocytosis. The Rho GTPases are regulated by guanine nucleotide exchange factors (GEFs) and GTPase-activating proteins (GAPs) [3]. GEFs catalyze the conversion from GDP-bound to GTP-bound Rho forms while RhoGAPs stimulate the weak intrinsic GTP-hydrolysis activity of Rho GTPases.

The human genome encodes about 80 RhoGAPs that regulate the 20 known Rho GTPases [4]. The function and regulation of RhoGAPs are controlled through multiple mechanisms including phosphorylation, protein–protein interactions, lipids and protein degradation.

**Figure 1:**
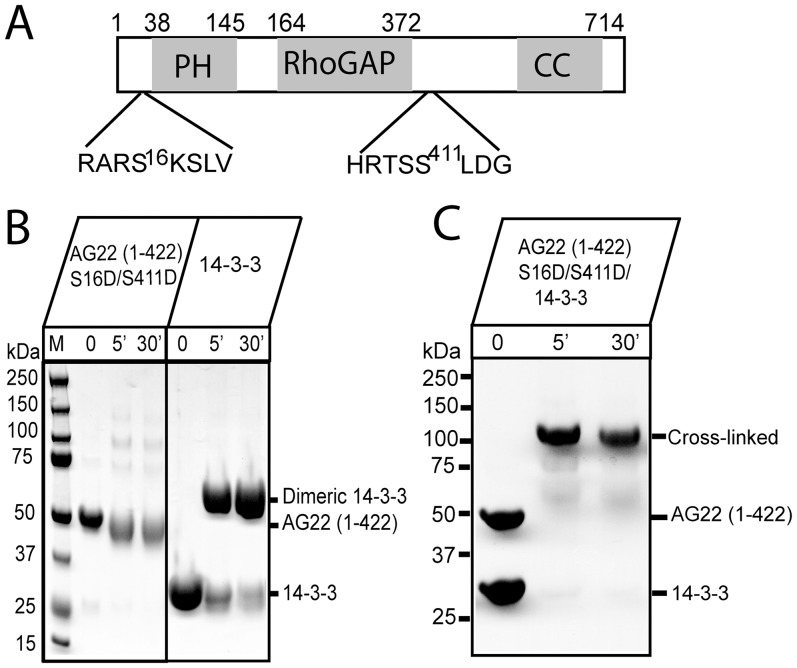
Cross-linking of ARHGAP22 and 14-3-3. **A.** Domain organisation of human ARHGAP22 (AG22), indicating the PH, RhoGAP and coiled-coil domains, as well as the two Akt phosphorylation sites (Ser16 and Ser411). **B.** AG22 (1–422) S16D/S411D and human 14-3-3 were individually incubated with BS3 for 0, 5 or 30 min at room temperature before separation of the cross-linked components on SDS-PAGE, showing that AG22 (1–422) S16D/S411D is monomeric and 14-3-3 is dimeric (monomer MW, 29.3 kDa). **C.** A mixture of AG22 (1–422) S16D/S411D and human 14-3-3 was incubated with BS3 for 0, 5 or 30 mins at room temperature before separation on SDS-PAGE. For both panels B and C, the position of bands corresponding to the untreated proteins and cross-linked product are indicated on the right. Molecular weights (kDa) of markers are indicated on the left. Cross-linking experiments shown are representative of three replicates.

ARHGAP22 (designated here as AG22) (gene *RhoGap2*) belongs to the RhoGAP family. The AG22 protein is comprised of an N-terminal pleckstrin homology (PH) domain, preceding a RhoGAP domain and followed by a C-terminal coiled-coil domain (Figure 1A). Recent studies have demonstrated that AG22 is a key regulator affecting modes of cancer cell movement through its ability to inactivate Rac [5]. Moreover, AG22 is an insulin-responsive and Akt-dependent 14-3-3 binding protein [6].

The 14-3-3 protein family has seven human isoforms (designated β, ε, γ, η, σ, τ, ζ) that are highly conserved and ubiquitously expressed [7]. 14-3-3 proteins primarily interact with phospho-serine/threonine binding proteins containing either a mode 1 motif (RSXpSXP) or a mode 2 motif (RXF/YXpSXP, where pS represents phosphoserine or phosphothreonine and X represents any amino acid) [8], [9]. The phospho-specific binding motif is required for high affinity binding of 14-3-3 to the phosphorylated protein [7]. However, it is noteworthy that 14-3-3 proteins can also interact with non-phosphorylated binding partner proteins [10]. 14-3-3 proteins primarily function as dimers with two binding pockets for target proteins [9].

Proteomic analysis previously identified a number of 14-3-3-interacting proteins involved in cytoskeletal regulation and GTPase function, including 14 GTPase regulatory proteins [11]. ARHGAP22 was found to bind to the 14-3-3 β isoformin response to insulin signalling [12]. Recent findings suggested that AG22 contains two potential 14-3-3 binding sites at residues Ser16 and Ser411 (equivalent to Ser395 in the sequence numbering for isoform 3, comprising 698 residues), which mediate insulin-stimulated 14-3-3 binding [6]. Indeed, motif analysis by SCANSITE [13] predicts two Akt-dependent phosphorylation sites at residues Ser16 (RARS^16^KSLV) and Ser411 (HRTSS^411^LDG). On the basis of SCANSITE scoring, these two residues matched medium-stringency Akt phosphorylation sites (RXRXXS/T) [14]. Ser16 is also a medium-stringency 14-3-3 binding site and Ser411 is a low-stringency 14-3-3 binding site (ie these residues fall in the top 1% and 5% cut-off for predicted 14-3-3 binding, respectively). Subsequent functional studies showed that Ser16 is a phosphorylation target of the kinase Akt [6].

Here, we show that a truncated form of AG22 interacts directly with 14-3-3, with a binding stoichiometry of one AG22 molecule per 14-3-3 dimer. The complex is weak (∼30 µM) and can be stabilized and detected by chemical cross-linking, which we used in combination with small angle X-ray scattering to determine the binding affinity and solution structure of the AG22:14-3-3_2_ complex.

## Results

We were unable to produce full-length human isoform 1 of AG22 (residues 1–714) in a stable purified form. Thus, we used a shortened construct AG22 (residues 1–422) which includes the PH and RhoGAP domains and the two phosphorylation sites, Ser16 and Ser411. We also generated constructs containing only the PH domain (residues 1–145, AG22 (1–145)), the RhoGAP domain (residues 164–422, AG22 (164–422)). Additionally, we created an AG22 (1–422) phosphomimic mutant where Ser16 and Ser411 were replaced with aspartates AG22 (1–422) S16D/S411D.

### AG22 (1–422) S16D/S411D and 14-3-3 form a weak or transient complex

We first investigated the interaction using a GST pull-down approach whereby GST-tagged AG22 (1–422) S16D/S411D phosphomimic double mutant was immobilized on glutathione beads, incubated overnight with human 14-3-3 β (referred to hereafter as 14-3-3) then washed extensively, but we were unable to detect an interaction (Figure S1). However, small angle X-ray scattering titration experiments did indicate that an interaction occurs between these two proteins, (Figure S2). Reasoning that the interaction might be weak or transient, we then used cross-linking to investigate the interaction further, because cross-linking can stabilize and detect weak/transient interactions when other techniques fail [15]. We used the chemical cross-linker bis(sulfosuccinimidyl) suberate (BS3) which, with high specificity, cross-links lysine amines with a spacer length of 11.4 Å.

We first analysed the individually purified proteins. Addition of BS3 to AG22 (1–422) S16D/S411D phosphomimic double mutant resulted in a single band on SDS-PAGE of molecular mass ∼50 kDa (calculated monomer mass, 49.7 kDa), indicating that the protein is monomeric in solution (Figure 1B). By contrast, incubation of 14-3-3 with BS3 resulted in a major band on SDS-PAGE of molecular weight ∼60 kDa (Figure 1B) corresponding to the size of an intra-molecularly cross-linked 14-3-3 dimer (calculated monomer mass, 29.3 kDa).

Cross-linking of a mixture of AG22 (1–422) S16D/S411D and 14-3-3 resulted in a major band on SDS-PAGE, indicative of a cross-linked product with a molecular mass of ∼100 kDa (Figure 1C). The size of the cross-linked complex is consistent with an interaction of one AG22 (1–422) S16D/S411D molecule (49.7 kDa) with a 14-3-3 dimer (58.6 kDa).

These results show that the AG22 (1–422) construct we generated is able to interact directly, if weakly or transiently, with human 14-3-3. The stoichiometry of the interaction identified by cross-linking is consistent with literature reports [9] that 14-3-3 proteins exist as dimers and interact as dimers with their target proteins. We also found that wild type AG22 (1–422) lacking the phosphomimic mutations can also be cross-linked with 14-3-3 with the same 1∶2 stoichiometry (Figure S3), suggesting that the interaction of AG22 with 14-3-3 may be independent of phosphorylation.

### Size-exclusion chromatography confirms a weak or transient complex

**Figure 2:**
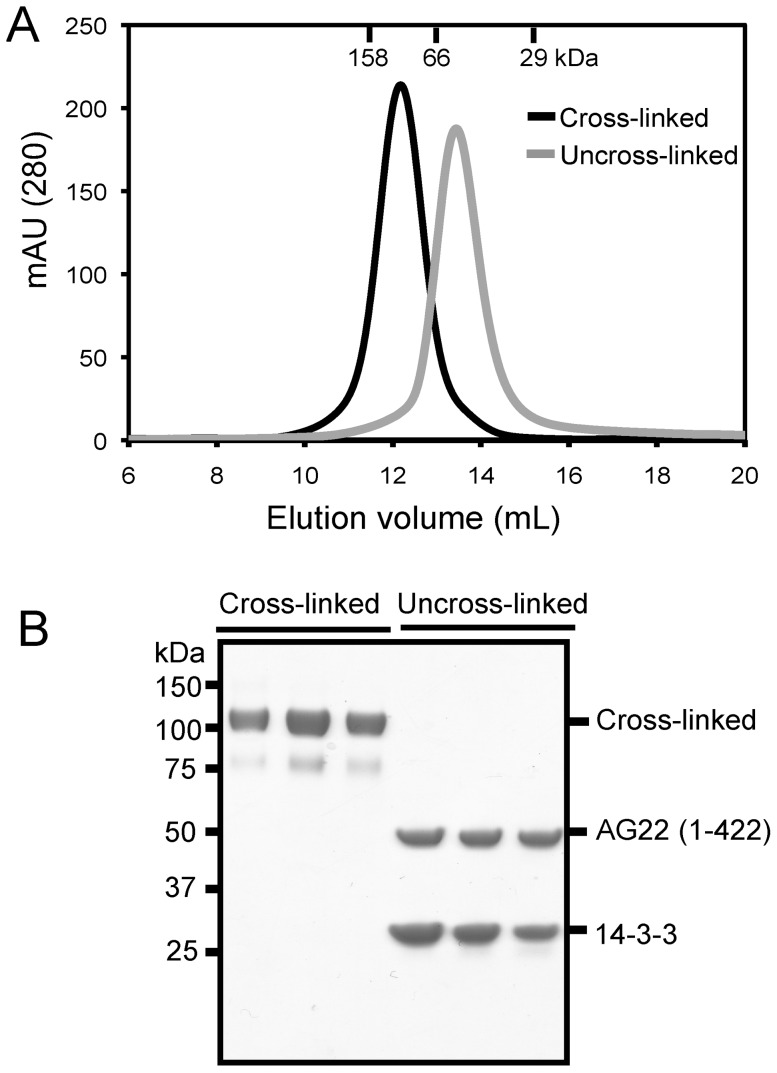
SEC analysis of AG22 (1–422) S16D/S411D and 14-3-3. **A.** SEC elution profiles of the cross-linked (black) and uncross-linked (gray) complexes. The cross-linked complex of AG22 (1–422) S16D/S411D and 14-3-3 eluted at 12 mL from a Superdex S200 (10/300 GL) column, while the uncross-linked complex eluted at 13.5 mL. Absorbance (mAU) at 280 nm was monitored. Elution volumes of molecular mass standards are indicated at the top of the panel. **B.** Elution fractions from gel filtration were analyzed by SDS-PAGE and stained with Coomassie Blue.

To further characterise the nature of the interaction, we used size exclusion chromatography (SEC) of AG22 (1–422) S16D/S411D and 14-3-3 before and after cross-linking. A mixture of AG22 (1–422) S16D/S411D phosphomimic double mutant and 14-3-3 at a 1∶2 molar ratio was cross-linked with BS3 as described above and the cross-linked products purified by SEC. A 1∶2 mixture of the same two purified proteins without cross-linking was also characterised by analytical SEC (S200 10/300GL, total volume 24 mL). Figure 2 shows that the cross-linked complex elutes differently to the uncross-linked complex. The cross-linked complex elutes at 12 mL, corresponding to an apparent molecular size of ∼100 kDa, consistent with a 1∶2 stoichiometry of AG22 (1–422) S16D/S411D and 14-3-3. However, without cross-linking the two proteins elute later, at 13.5 mL, consistent with a molecular size of less than 66 kDa. This result indicates that without cross-linking the 14-3-3 dimer (2×29.3 kDa) and the AG22 (1–422) S16D/S411D monomer (49.7 kDa) co-elute on SEC because of their similar masses, but do not form a stable complex (a stable complex would elute at ∼12 ml, similar to that of the cross-linked complex).

The SEC data support the notion that AG22 (1–422) S16D/S411D and 14-3-3 do not form a stable complex under the conditions we used, but that cross-linking can stabilise a weak or transient complex of the two proteins that can then be eluted as a single monodisperse peak with a 1∶2 stoichiometry.

### Interaction with 14-3-3 requires the PH domain of AG22

**Figure 3:**
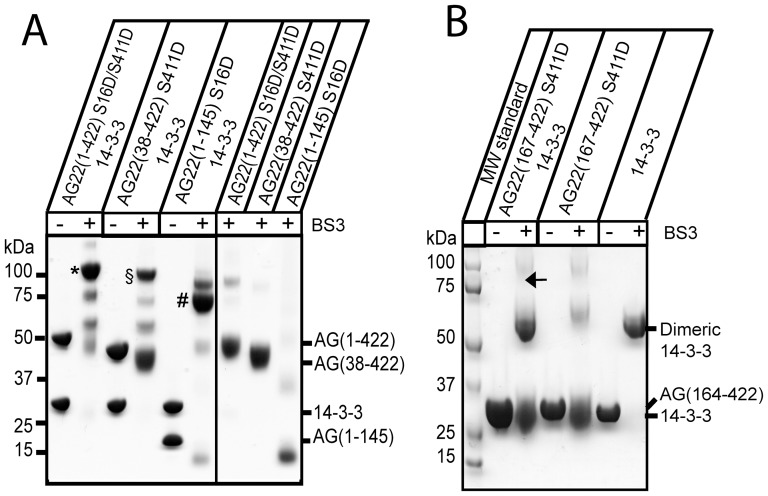
Cross-linking of AG22 constructs with 14-3-3. **A.** Cross-linking of truncation constructs AG22 (1–422) S16D/S411D, AG22 (38–422) (S411D) and AG22 (1–145) S16D with 14-3-3. For each cross-linking reaction, the mixture of AG22 protein construct and 14-3-3 or the individual AG22 proteins was incubated with BS3 for 10 min at room temperature. The mixtures before and after cross-linking were analyzed by SDS-PAGE and visualized by Coomassie Blue staining. Lanes on the left show BS3 cross-linking results with 14-3-3; lanes on the right show BS3 cross-linking results for AG22 proteins without 14-3-3. *indicates the cross-linked complex of 14-3-3 with AG22 (1–422) S16D/S411D; ^§^the cross-linked complex of 14-3-3 with AG (38–422) S411D and ^#^the cross-linked complex of 14-3-3 with AG22 (1–145) S16D. **B.** No detectable cross-linking was observed for AG22 (167–422) S411D and 14-3-3. The arrow indicates the position of the band (90 kDa) expected if AG22 (164–422) S411D (MW, 31.3 kDa) and 14-3-3 (monomer MW, 29.3 kDa) had formed a cross-linked complex. Cross-linking experiments shown in panels A and B are representative of three replicates.

To further probe the regions of AG22 contributing to 14-3-3 interaction, we generated purified forms of three additional AG22 constructs: AG22 (38–422) S411D (comprising the PH and RhoGAP domain and the C-terminal phosphomimic mutation, but lacking the N-terminal 37 residues); AG22 (1–145) S16D (comprising the PH domain and the N-terminal phosphomimic mutation, but lacking the RhoGAP domain and the C-terminal phosphomimic mutation); and AG22 (164–422) S411D (comprising the RhoGAP domain and C-terminal phosphomimic mutation, but lacking the PH domain and N-terminal phosphomimic mutation). Subsequently, we performed 14-3-3 cross-linking reactions using BS3 as described above. Under the same conditions, AG22 (38–422) S411D produced considerably less cross-linked complex with 14-3-3 compared with AG22 (1–422) S16D/S411D (Figure 3A). By contrast, AG22 (1–145) S16D (which contains only the PH domain and the N-terminal phosphomimic mutation) was almost completely cross-linked to 14-3-3 (Figure 3A). The major cross-linked product migrates on SDS-PAGE at ∼80 kDa consistent with a 1∶2 stoichiometry of AG (1–145) S16D (19.2 kDa) and 14-3-3. The cross-linked product was also confirmed by SEC analysis (Figure S4).

However, cross-linking did not detect an interaction between AG22 (164–422) S411D and 14-3-3 (Figure 3B). If these two proteins had been cross-linked, a band corresponding to a mass of ∼88 kDa would be expected, and no such band was present. These results indicate that the RhoGAP domain alone is not sufficient for AG22 binding to 14-3-3, but that the PH domain with the N-terminal Ser16 phosphomimic mutation is sufficient for 14-3-3 binding to be detected by cross-linking.

### MS identification of cross-linked peptides supports a role for the PH domain

To obtain more detail about the regions involved in the interaction, we used liquid chromatography coupled with electrospray ionization mass spectrometry (LC-ESI-MS) to analyse the cross-linked complex of AG22 (1–422) S16D/S411D and 14-3-3. Thus, the covalently cross-linked complex was purified by SEC and subsequently subjected to trypsin digestion and LC-ESI-MS analysis. We used the xQuest [16] web-server to generate possible cross-links and manually verified the assigned peptide sequences by inspection of the raw ESI-MS spectra. Details of the confirmed cross-linked peptides (four intermolecular and one intramolecular) are provided in Table 1. The three lysine residues identified by LC-ESI-MS as being involved in cross-linking (K15, K45 and K52) are all located at the N-terminal and PH domain of AG22. No cross-links were detected between the RhoGAP domain and 14-3-3, in agreement with the domain analysis results described above. (The PH and RhoGAP domains of AG22 contain 11 and 8 lysines, respectively).

**Table 1:** MS identification of peptide sequences cross-linked in the AG22 (1–422) S16D/S411D:14-3-3 complex.

Observed mass	Predicted mass	Assigned peptide sequences	Cross-links
1557.9	1557.8	AGWLK_45_K-K_77_QQMGK	AG22:14-3-3
2128.4	2128.1	AGWLK_45_K-VISSIEQK_70_TER	AG22:14-3-3
2255.2	2255.2	SIMK_52_NWQQR-VFYLK_122_MK	AG22:14-3-3
2675.6	2675.3	SK_15_DLVMGEQSR-VISSIEQK_70_TER	AG22:14-3-3
5313.8	5313.6	QTTVSNSQQAYQEAFEISK_159_K- LGLALNFSVFYYEILNSPEK_189_ACSLAK	14-3-3:14-3-3

### SAXS data confirm a role for PH but not RhoGAP domain interacting with 14-3-3

To determine the low-resolution solution structure of the complex, we used small angle X-ray scattering (SAXS). We measured data for 14-3-3 alone, AG22 (1–422) S16D/S411D alone, as well as the purified cross-linked complex (Table S1).

**Figure 4:**
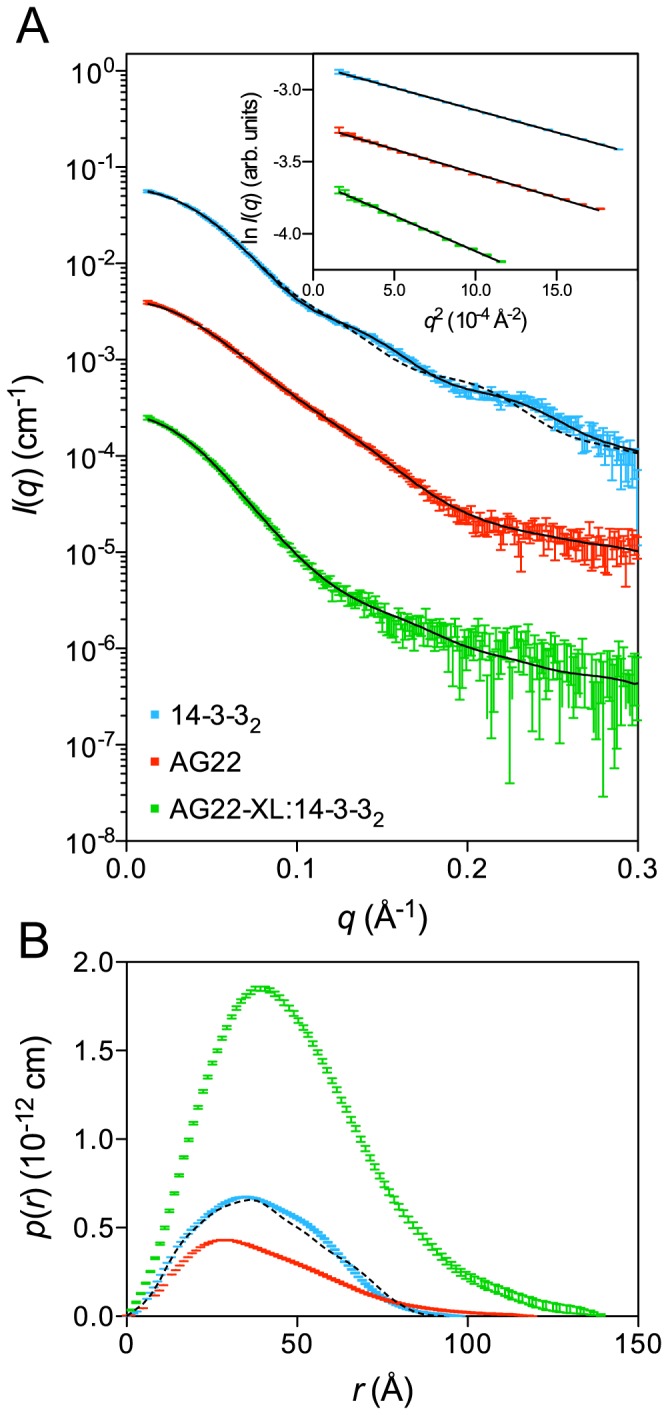
Small angle X-ray scattering data for AG22 (1–422) S16D/S411D and 14-3-3. **A.** The measured scattering data for 14-3-3 (blue), AG22 (1–422) S16D/S411D (red – offset by a factor of 10^−1^), purified cross-linked AG22 (1–422) S16D/S411D:14-3-3_2_ complex (green – offset by a factor of 10^−2^) overlaid with the SAXS profiles of the optimized models (solid black lines: *χ* ^2^(14-3-3) = 1.3; *χ* ^2^(AG22 (1–422) S16D/S411D) = 1.1; *χ* ^2^(AG22 (1–422) S16D/S411D:14-3-3_2_ cross-linked complex) = 0.9). Also overlaid is the predicted scattering profile for the 14-3-3 crystal structure (black dotted line: *χ* ^2^(PDB: 2BQ0) = 7.4). Inset: Guinier plots of the low-angle portion of the scattering data are linear consistent with monodisperse solutions. **B.** Pair-distance distribution functions derived from the scattering data using GNOM [31]. The predicted *p*(*r*) profile for the 14-3-3_2_ crystal structure (black dotted line) is shown for comparison. Colouring scheme is the same as for panel A. This shows as expected that the complex is larger than the AG22 (1–422) S16D/S411D or 14-3-3 molecules on their own.

The Guinier region of the 14-3-3 scattering data is linear (Figure 4A, inset) and the molecular mass calculated from the scattering data is 55 kDa, in good agreement with the calculated mass of a 14-3-3 dimer (58.6 kDa) [9], indicating that the sample was monodisperse. The *p*(*r*) is also consistent with a 14-3-3 dimer, showing a maximum dimension of 100 Å (larger than the 70 Å expected for a 14-3-3 monomer, and similar to the 95 Å expected for 14-3-3 dimer) and displaying a shoulder at ∼60 Å, consistent with a dimeric arrangement. The predicted scattering profile calculated from the 14-3-3 crystal structure fits the scattering data reasonably well with *χ* ^2^ = 7.4 and a radius of gyration (*R*_g_) of 30.7 Å (compared to the experimental value of 30.4 Å). However, there are systematic differences between the measured scattering data and the predicted scattering profile (Figure 4A). To investigate the nature of these differences, a structural model of 14-3-3 was optimised against solution scattering data using the program BUNCH [17] (Figure S5A). The optimised model provides an excellent fit to the scattering data (Figure 4A, *χ* ^2^ = 1.3), and is very similar to the 14-3-3 crystal structure with an RMSD of 2.05 Å between the two structures. The most significant difference between the crystal structure and the model optimised against the scattering data is a change in the orientation of the C-terminal portion of 14-3-3 (residues 167–232). While the model provides an excellent fit to the data, this change may reflect flexibility in this region rather than a specific conformational change.

The Guinier region of the AG22(1–422) S16/S411D scattering data is also linear (Figure 4A, inset), and the calculated molecular mass of 45 kDa is in good agreement with the expected value of 47.2 kDa for the TEV-cleaved AG22(1–422) S16/S411D protein, indicating that the samples are monodisperse. The shape of the *p*(*r*) reveals the structure is extended (Figure 4B), with a maximum dimension of ∼120 Å and *R*_g_ of 31.4 Å. Rigid body modelling of AG22 (1–422) S16D/S411D provided an excellent fit to the scattering data (Figure 4A, *χ* ^2^ = 1.1) and suggests that in solution the PH and RhoGAP domains are not “beads-on-a string” domains separated by large distances. Rather, the modelling indicates that the domains are in reasonably close proximity to each other with a linker region (residues 146–163) that is likely to be structured (Figure S5B). However, modelling against the SAXS data does suggest that the N- and C-terminal regions of AG22 (1–422) S16D/S411D that contain the phosphomimic aspartate mutations S16D and S411D are extended and unstructured in solution.

**Figure 5:**
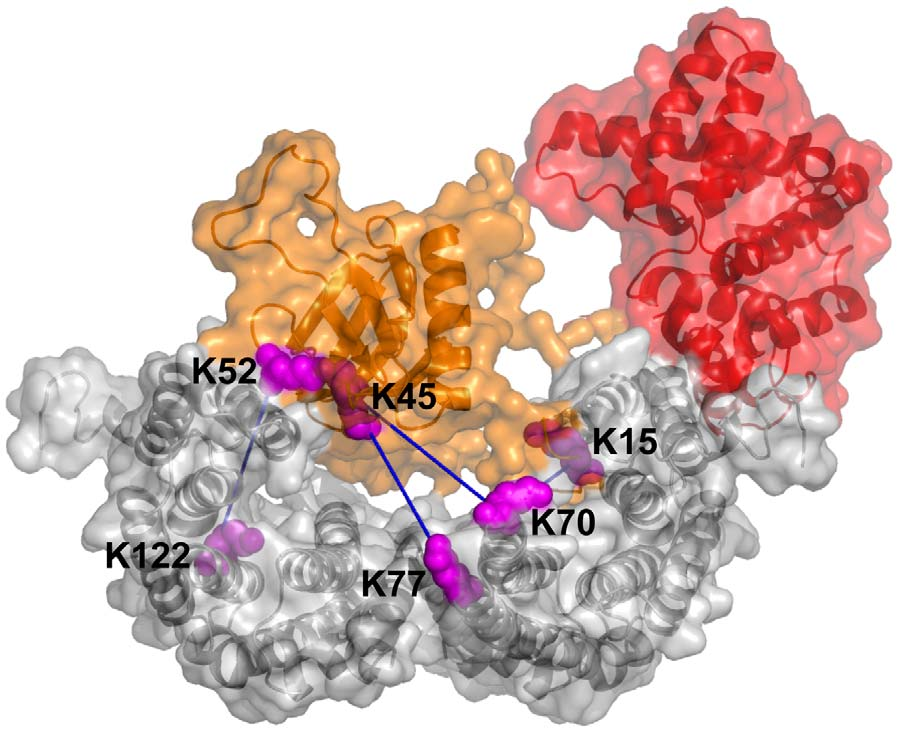
Modeled solution structure of the cross-linked complex between AG22 (1–422) S16D/S411D and 14-3-3. The model of the cross-linked complex is shown with the 14-3-3 dimer in grey, the AG22 PH domain (residues 1–145) in orange and the AG22 linker and RhoGAP domain (146–372) in red. For clarity, the C-terminal residues 373–422 of AG22 are not shown. The intermolecular cross-links between AG22 (1–422) S16D/S411D and 14-3-3 are indicated with dark blue lines: these are K15^AG^:K70^14-3-3^, K45^AG^:K70^14-3-3^, K45^AG^:K77^14-3-3^ and K52^AG^:K122^14-3-3^. The cross-linked lysine sidechains are shown in magenta. The figure was generated using PyMol [33].

Preliminary analysis of the scattering data measured for the purified AG22 (1–422) S16D/S411D:14-3-3_2_ cross-linked complex again showed a linear Guinier region (Figure 4A, inset), with a calculated molecular mass of 105 kDa, in good agreement with the expected value of 105.8 kDa. The shape of the *p*(*r*) reveals a more globular structure than for AG22 (1–422) S16D/S411D, but the profile slowly approaches ∼145 Å, indicating that part of the structure is relatively extended. Structural modeling of the cross-linked complex consistently provided models compatible with the SAXS data when the N-terminal phosphomimic site (S16D) was restrained in the peptide-binding pocket of the 14-3-3 structure, and the PH domain was restrained to make contact with the 14-3-3 dimer (the restraints used were the sequenced chemical cross-links, Table 1). Attempts to include an additional interaction between the C-terminal AG22 phosphomimic site S411D and the second peptide-binding pocket of 14-3-3 did not produce a model consistent with the SAXS data. The resulting models of the complex show the AG22 PH domain interacting with 14-3-3 in a reasonably well-defined position. The position of this PH domain is determined by both the cross-link restraints and scattering data, and occupies a sufficient portion of the 14-3-3 surface to prevent the binding of a second AG22 molecule to the 14-3-3 dimer, explaining the observed stoichiometry. The AG22 RhoGAP domain is located at different positions around the periphery of the 14-3-3 dimer in each model. However, the overall shape of each model of the complex is roughly similar, explaining why the scattering data appear to be insensitive to the position of the RhoGAP domain. The four best models of the complex (assessed on *χ* ^2^) show the RhoGAP domain at the four corners of the 14-3-3 dimer (Figure S5C), each with a different distance to the corresponding PH domain. As a result of this variability we cannot determine if there are any structural rearrangements of AG22 upon 14-3-3 binding in the absence of other complementary structural or biochemical data. The model providing the best fit to the scattering data (Figure 5) is an excellent fit (Figure 4A, *χ* ^2^ = 0.9) and is representative of the models obtained. The position of the PH domain is consistent with the sequenced chemical cross-links (Table 1), where the RhoGAP domain projects away from the PH domain making few interactions with 14-3-3.

The modelled position of the N-terminal region of AG22 (1–422) S16D/S411D in the 14-3-3 binding pocket is consistent with a confirmed sequenced cross-link between residue Lys15 of AG22 (1–422) S16D/S411D and Lys70 of 14-3-3. However, the cross-linking data also suggest that both the native AG22 (1–422) and the AG22 (1–422) S16D/S411D phosphomimic double mutant bind 14-3-3. Hence, it is possible that this N-terminal peptide could have a different 14-3-3 binding mode to that of the canonical phospho-peptide interaction described in several 14-3-3 crystal structures, and which we have used in modelling the interaction. As the scattering data yields low-resolution information, it is not sensitive to details such as the binding mode of a small peptide in the context of a large protein complex. Nevertheless, the shape of the modelled complex is consistent with the scattering data; the modelled arrangement of domain interactions is consistent with sequenced cross-links and with cross-linking of 14-3-3 with AG22 domain constructs, showing that the RhoGAP domain of AG22 does not make significant interactions with 14-3-3 under the conditions we used.

### AG22 (1–422) S16D/S411D binds to 14-3-3 with micromolar affinity

To probe the affinity of the interaction between AG22 (1–422) S16D/S411D and 14-3-3, small-angle scattering data was measured on samples containing AG22 (1–422) S16D/S411D at a concentration of 1.25 mg/mL (26.5 µM) and 14-3-3_2_ at 0.5, 1.0, 1.5 and 2.0 times the molar concentration of AG22 (1–422) S16D/S411D. The Guinier region of the scattering data collected from each sample was linear, indicating monodisperse solutions. For an equilibrium solution such as this, the scattering will be a linear combination of scattering from 14-3-3_2_, AG22 (1–422) S16D/S411D, and AG22 (1–422) S16D/S411D:14-3-3_2_. We fitted a linear combination of the scattering curves for the isolated proteins to each data set, constrained by the concentrations of AG22 (1–422) S16D/S411D and 14-3-3. The fit to the scattering data was very good in each case (Figure S2). The calculated dissociation constants at each molar ratio were in good agreement (Table S2), yielding an average *K*_d_ value of 28±6 µM. We were fortunate that the *K*_d_ value for the complex was of the same order as the concentrations used in each measurement, which meant that there was a significant amount of each component present. If the *K*_d_ value was orders of magnitude different to the protein concentrations used, the method would be far less accurate as the amount of reactants or products would be small. This approach is also dependent on having a scattering profile for the complex, in this case provided by the cross-linked complex.

### AG22 (1–422) does not interact with Rac

AG22 has been associated with the switching of tumor cells between rounded and elongated modes of motion, through inactivation of the GTPase Rac [5]. Several other GTPase-activating proteins (GAPs) have been found to form complexes with the hydrolysis-impaired Q61L mutant of Rac [18]. Moreover, the addition of aluminium fluoride - that mimics the γ-phosphate of GTP - has been used to induce complex formation between GDP-bound Rac (Rac.GDP) and GAPs [19]. We therefore attempted to isolate the complex of AG22 (1–422) and other AG22 truncations with Rac1:GDP or Rac Q61L using cross-linking and SEC. However, we were unable to detect complex formation (Figures S6 and S7).

## Discussion

Structural and functional analysis of protein-protein interactions *in vitro* requires the formation of stable and homogenous protein complexes. The 14-3-3 protein has been shown to interact with many proteins [11], [20], so we engineered constructs of AG22 that could be produced and purified for interaction studies with 14-3-3. We were unable to generate full-length AG22 because it was unstable and readily degraded. We therefore used a truncated construct AG22 (1–422) that lacked the C-terminal coiled coil domain, but included the PH and RhoGAP domains and two phosphorylatable serine residues (Ser16 and Ser411) identified as potential binding sites for 14-3-3 [6]. We also generated a phosphomimic double mutant of this construct, AG22 (1–422) S16D/S411D, in which the two serine residues were replaced with negatively charged aspartate residues. Our attempts to produce the complex of AG22 (1–422) S16D/S411D with 14-3-3 using pull-down assays, SEC and co-purification were unsuccessful. However, titration of 14-3-3 into AG22 (1–422) S16D/S411D and analysis by SAXS did indicate an interaction occurs, and yielded a relatively weak dissociation constant (*K*_d_) of ∼30 µM, explaining the difficulty we had in isolating the complex using affinity resin or SEC. The use of SAXS to quantify the affinity is a novel application of this technique and may be pertinent to other weakly interacting systems where the same conditions apply (*ie* concentrations of the components in the titration series are similar to the *K*_d_; SAXS data for individual components and cross-linked complex are available).

Chemical cross-linking is a powerful method for detecting transient complexes because this approach can stabilize weak interactions [15], and was of critical importance to the success of this work. Chemical cross-linking of AG22 (1–422) S16D/S411D and 14-3-3 clearly showed that these two proteins are able to interact directly, albeit weakly. Furthermore, SEC and SDS-PAGE analysis of the cross-linked complex indicated that the binding stoichiometry is one AG22 monomer per 14-3-3 dimer. We also used small angle X-ray scattering from the cross-linked complex to generate a model of the solution structure of the AG22:14-3-3_2_ complex. Although many crystal structures of 14-3-3 bound to peptide ligands have been determined, there is only one crystal structure of 14-3-3 bound to a target protein, that of serotonin N-acetyltransferase (AANAT) [21], [22]. Thus, our structure of AG22:14-3-3_2_ provides new information on the way in which 14-3-3 can interact with partner proteins, in this case showing that a weak or transient interaction occurs when one rather than two of the 14-3-3_2_ peptide binding sites is occupied. This weak interaction was captured by chemical cross-linking.

Why is the AG22 complex with 14-3-3 relatively weak? One possibility is that a weak or transient interaction of these two proteins is required in the context of cellular function. Analysis of phosphorylation effects on protein-protein interactions in the human proteome, showed that phosphorylation sites are often located on binding interfaces in weak or transient complexes and for the majority of complexes, phosphorylation was predicted to have minimal effect on stability and binding affinity [23]. An example of another weak interaction involving 14-3-3 is the interaction with AS160 [24]. AS160 is also an Akt substrate, a RabGAP that mediates insulin-stimulated GLUT4 translocation. Phosphorylation of AS160 by PKB/Akt leads to 14-3-3 binding. However the binding affinity of AS160 to 14-3-3 is weak and the complex dissociates during immunoprecipitation wash steps [24]. The weak/transient interaction between AS160 and 14-3-3 was also stabilized by chemical cross-linking allowing detection by co-immunoprecipitation from cell lysates [24].

A weak or transient interaction between AG22 and 14-3-3 may thus be a characteristic of the complex *in vivo*, or perhaps additional partner proteins may be required to reinforce the interaction. Alternatively, a strong interaction may be necessary *in vivo*, but may not be generated under the experimental conditions we used. For example, if the coiled coil domain is important for the 14-3-3 interaction, then removing it to produce the soluble, stable form of the AG22 protein that we used might reduce 14-3-3 binding affinity. Similarly, the aspartate mutants we used to mimic phosphorylated AG22 may not be optimal mimics of phosphorylation in this system. However, the fact that native AG22 (1–422) can also be cross-linked to 14-3-3 suggests that AG22 phosphorylation (or at least modification of serine to a negatively charged residue) is not an essential component of the 14-3-3 interaction. Indeed, there are precedents for phosphorylation-independent binding of 14-3-3 to target proteins (*eg* the 14-3-3 binding peptide of the C-terminal domain of p190RhoGEF has no serine or phosphoserine, it's sequence is I^1370^QAIQNL) [25].

We found that the RhoGAP domain of AG22 was not sufficient to interact with 14-3-3. This conclusion is supported by recent findings that binding of 14-3-3 to the pSer411 binding site (C-terminal to the RhoGAP domain) is dependent on binding to pSer16 preceding the PH domain [6].

Although AG22 has been identified as an important binding partner of Rac1 [5], we were unable to detect an interaction between the two, even with cross-linking. This interaction may therefore require the C-terminal coiled-coil domain of AG22, because full-length AG22 was used in the *in vivo* binding studies [5]. As described above, we were unable to use recombinant full-length AG22 due to rapid degradation. It is also possible that additional post-translational modifications or regulatory proteins are required for binding of AG22 to Rac1; these would be present *in vivo* but not in our *in vitro* experiments. It is noteworthy that the intrinsic GTPase activity of FilGAP, a related GAP protein which like AG22 has PH, GAP and coiled-coil domains, requires phosphorylation by ROCK [26].

In summary, we have shown that the PH/RhoGAP region of AG22 interacts with 14-3-3 with a binding affinity of ∼30 µM, that the PH domain alone is sufficient for this weak interaction, but that neither of these AG22 constructs interacts with Rac1. A low-resolution solution structure of the AG22 (1–422) S16D/S411D:14-3-3_2_ complex provides a model for the interaction and suggests that one rather than the usual two binding sites of 14-3-3 is occupied by AG22 peptide loops. More broadly, our work has demonstrated the power of combining the hybrid techniques of chemical cross-linking, ESI-MS, and SAXS to obtain important new structural insights into weak and transient protein complexes.

## Materials and Methods

Unless otherwise noted, chemicals were obtained from Sigma.

### Cloning of AG22 and 14-3-3 constructs

Constructs AG22 (1–422), AG22 (38–422), AG22 (1–145) and AG22 (164–422) of human ARHGAP22 (isoform 1, 714 residues) (Open Biosystems, Accession number 12644; GenBank: AAI26445) were subcloned into the LIC vector pMCSG7 [27] encoding an N-terminal polyhistidine tag with a tobacco etch virus (TEV) cleavage site. The double mutant S16D/S411D of AG22 (1–422) was generated using the Quikchange II XL site-directed mutagenesis kit following manufacturer's instructions (Stratagene, USA). The codon-optimized synthetic gene of human 14-3-3 β isoform (GeneArt) was subcloned into pET20b encoding a C-terminal hexa-histidine tag. Sequences of all DNA constructs were confirmed by DNA sequencing. The codon-optimized synthetic gene of human Rac1 (GeneArt) was subcloned into pMCSG7 encoding an N-terminal His tag and a TEV protease cleavage site. The AG22 (1–422) construct was subcloned into pMCSG10 which encodes an N-terminal His tag, followed by glutathione-S-transferase (GST) and a TEV protease cleavage site. The Rac1 mutant Q61L was generated using the Quikchange II XL site-directed mutagenesis protocol (Stratagene, La Jolla, CA, USA).

### Protein Expression and Purification

Proteins were expressed in *E coli* BL21(DE3)pLysS at 25°C for 24 h by autoinduction [28]. Cells were lysed in 50 mM phosphate buffer pH 8.0 containing 300 mM NaCl, 10% glycerol, 1% Triton X-100, 12,500–14,000 U DNase (Sigma Aldrich, Roche), 100 µL of protease inhibitor cocktail III (AG Scientific Inc) and 2 mM β-mercaptoethanol (β-ME). Metal affinity purification was performed using Co^2+^-affinity beads (Clontech) for 14-3-3 or Ni-chelated PrepEase resin (USB Corporation) for AG22 constructs. Cleared lysate was incubated with resin for 0.5–1.5 h, washed first with 10 mM imidazole and then with 20 mM imidazole in wash buffer A (50 mM phosphate buffer pH 8.0, 300 mM NaCl, 10% glycerol, 2 mM β-ME). The purified protein was eluted with 300 mM imidazole in wash buffer. In the final purification step, protein samples were injected onto and eluted from a size exclusion chromatography XK16/60 column packed with Superdex S-200 (GE Healthcare) and equilibrated in buffer B (25 mM HEPES pH7.5, 150 mM NaCl, and 2 mM β-ME). To remove the engineered His-tag from AG22 proteins, resin-purified protein was incubated at 4°C overnight with His-tagged TEV protease. The protease and uncleaved AG22 were separated from cleaved AG22 using Co^2+^-affinity beads, and AG22 was further purified by size exclusion chromatography as described above.

To prepare GDP-bound Rac1, the cell pellet was resuspended in lysis buffer containing 25 mM Tris-HCl pH 7.5, 50 µM GDP, 300 mM NaCl, 10 mM MgCl_2_, 10% glycerol, 0.5% Triton X-100, 12,500–14,000 U DNase (Sigma Aldrich, Roche), 100 µL of protease inhibitor cocktail III (AG Scientific Inc) and 2 mM β-ME. In the case of Rac1 Q61L, GDP was not included in the lysis buffer.

### GST pull-downs

GST-ARHGAP22 (1–422) S16D/S408D was purified by glutathione affinity resin (GE Healthcare) and then incubated overnight at 4°C with purified His-tagged 14-3-3 in a total volume of 200 µL binding buffer containing 25 mM Tris-HCl, pH 7.5, 150 mM NaCl, 10% glycerol, 0.2% TX-100, 2 mM β-ME. The beads were washed extensively with binding buffer and bound proteins analysed on SDS-PAGE and Coomassie Blue.

### Chemical Cross-Linking

AG22 proteins (10 µM) and 14-3-3 (30 µM) either alone, or as a mixture in 50 mM HEPES, pH8.6 were incubated with a freshly prepared solution of 3 mM BS3 (Thermo Fisher Scientific) in 25 mM sodium phosphate buffer, pH 7.4 at room temperature for 5 or 30 min. The reaction was quenched by addition of 20 mM ammonium bicarbonate, pH 8.5 and the cross-linked products were analyzed by SDS-PAGE and Coomassie Blue staining.

### Size Exclusion Chromatography

A mixture of AG22 (1–422) phosphomimic mutant and 14-3-3 at a 1∶2 molar ratio was incubated with BS3 at room temperature for 30 min in 50 mM Hepes, pH 8.6. The reaction was quenched by addition of 50 mM Tris-HCl, pH 7.5. The cross-linked complex was then purified on a gel filtration Superdex S-200 XK16/60 column (GE Healthcare) equilibrated in buffer B. Analytical size exclusion chromatography was performed on a Superdex S200 10/300GL column (total column volume 25 mL) in buffer B. The uncross-linked complex of AG22 (1–422) S16D/S411D phosphomimic double mutant (4 mg/ml) and 14-3-3 (4.8 mg/ml) and the cross-linked complex (4 mg/ml) (total volume 250 µL) were loaded separately onto the column at a flow rate of 0.5 mL/min.

### SAXS Data Collection and Analysis

Data were collected on the SAXS-WAXS beamline at the Australian Synchrotron with a sample to detector distance set at 1567 mm and an X-ray wavelength of *λ* = 1.033 Å, allowing access to a *q*-range spanning ∼0.01–0.55 Å^−1^. Immediately prior to loading, all samples were centrifuged at 10,000 *g* to remove large particles from the solution. To minimise the effects of radiation damage, samples were flowed (50 µL each of 14-3-3, AG22 (1–422) S16D/S411D and the purified cross-linked complex) past the beam in 1.5 mm quartz capillaries (Hampton Research) at room temperature (295 K). Data were collected at a number of concentrations for each sample to determine the extent of concentration dependent attractive or repulsive interactions (1.50–5.80 mg/mL for 14-3-3; 1.25–2.60 mg/mL for AG22 (1–422) S16D/S411D; 0.15–0.35 mg/mL for the purified cross-linked complex). Titration experiments were performed by pre-mixing AG22(1–422) S16D/S411D at 26.5 µM (1.25 mg/mL) with 14-3-3 to give molar 14-3-3_2_:AG22 ratios of 0.5∶1.0, 1.0∶1.0, 1.5∶1.0 and 2.0∶1.0 (these complexes were incubated on ice for approximately 1 hour prior to centrifugation and measurement). For each sample, 5 frames (each with a 2 sec exposure time) were measured. Data reduction was carried out using SAXS15ID software [29], averaging all 5 measured frames and correcting for solvent scattering, sample transmission, detector sensitivity and background radiation. Data were placed on an absolute scale, by normalisation against a water standard.

Data quality was assessed by inspection of the concentration dependence of the scattering data, linearity of the Guinier region of the data, and estimated molecular mass of the protein complex. At all concentrations, the Guinier plot (ln *I*(*q*) *vs*. *q* ^2^) was linear, and based on the evolution of *I*(0) and *R*_g_ with concentration, it was deemed that inter-particle interactions are negligible below 3 mg/mL for all proteins (Table S1). Estimated molecular masses were determined for each protein complex (Table S1) using the equation described in [30] and were very close to the expected masses for each protein or protein complex. Taken together, these quality assessments indicate that the protein solutions used were homogeneous and free of significant inter-particle interactions, and the data measured were of high quality.

### Analysis of the SAXS titration data

Regularised scattering profiles for 14-3-3, AG22 (1–422) S16D/S411D, and cross-linked complex were generated using GNOM [31], and normalized by dividing the intensity data by the molar concentration of the sample (in the case of 14-3-3, the molar concentration was that of the dimer). Linear combinations of these profiles were fit to the measured scattering data, where the weight of each profile represents the concentration of that species in solution. The fit to the scattering data was optimized by varying the weights of each profile in a constrained manner, such that *W*_complex_ is the only free variable (*W*_Complex_ = [Complex]; *W*_AG22_ = [AG22]*_i_*−[Complex]; *W*_14-3-3_ = [14-3-3_2_]*_i_*−[Complex]). This was repeated for each titration point, providing a very good representation of the measured curves. The dissociation constant at each titration point was calculated as *K*_d_ = (*W*_AG22_×*W*_14-3-3_)/*W*_Complex_, and were all in good agreement allowing estimation of the *K*_d_ and the associated standard deviation.

### SAXS Data Modelling

The program BUNCH [17] is designed to model the structure of a single polypeptide chain, typically where structured regions are linked by flexible or unstructured regions. The program models regions of known structure as rigid units, and the remainder as chains of dummy residues. For the modelling of 14-3-3, the human 14-3-3 crystal structure (PDB ID: 2BQ0) [9] was divided into two domains corresponding to residues 3–161 and 167–232, with the 2-residue N-terminus, 13-residue C-terminus and 5-residue linker modeled with dummy residues. As the 14-3-3 crystal structure shows a dimer with *C*_2_ symmetry, the model was constrained to have the same symmetry. Structures for the PH (28–145) and RhoGAP (172–369) domains of AG22 were generated using I-Tasser [32], which uses structures deposited in the Protein Data Bank to build homology models based on sequence information. The confidence scores (c-score) of the best models were 0.32 and 1.10 for the PH domain (residues 28–145, 48% sequence identity to the model) and RhoGAP domain (residues 172–369, 26% sequence identity to the model), respectively. The program BUNCH was then used to optimise the position and relative disposition of each domain while representing the linker and unstructured regions with dummy residues.

The program CORAL [17] was used to model the 14-3-3_2_: AG22 (1–422) S16D/S411D complex. This program performs a rigid-body refinement of protein complexes, but instead of the approach used in the program BUNCH for representing flexible and unstructured regions, CORAL chooses from a library of linkers throughout the optimization. To generate the starting model, we took the 14-3-3 ζ bound peptide of serotonin N-acetyltransferase from the crystal structure of 14-3-3 complexed with serotonin N-acetyltransferase [21] (PDB ID: 1IB1) and mutated it to the AG22 peptide 11-RARSKDLV-18 (S16D phosphomimic mutant). During the optimization of the model of the complex against the SAXS data, the structure of 14-3-3 was fixed to that observed in the crystal structure (PDB ID: 2BQ0), and the position of the AG22 peptide (11–18) was fixed within the 14-3-3 peptide binding groove (as observed in 14-3-3_2_:serotonin N-acetyltransferase crystal structure). The positions of the AG22 PH and RhoGAP domains, and linker regions were optimised. Four sequenced cross-links between AG22 (1–422) S16D/S411D and 14-3-3 (K15^AG^:K70^14-3-3^, K45^AG^:K70^14-3-3^, K45^AG^:K77^14-3-3^ and K52^AG^:K122^14-3-3^) were included as restraints in the optimisation (K_Cα_–K_Cα_<30 Å). In parallel a similar optimisation was performed, where both the N-terminal and C-terminal phosphorylation mutation sites of AG22 were constrained to interact with the 14-3-3 peptide binding groove, but this gave unsatisfactory fits to the scattering data.

Ten models were generated for each structure, with the representative structure chosen on the basis of the statistic,
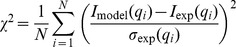
_._

### In-Solution Digestion and Mass Spectrometry Analysis

The BS3 cross-linked complex of AG22 (1–422) S16D/S411D and human 14-3-3 was purified by SEC as described above. The cross-linked complex was then denatured in 6M guanidine HCl, 50 mM NH_4_HCO_3_, pH 7.8, and reduced and alkylated with 5 mM DTT and 50 mM iodoacetamide (IAA) for 30 min at room temperature in the dark. The alkylated sample was buffer-exchanged into 50 mM NH_4_HCO_3_ and digested with trypsin (Promega sequencing grade) at 37°C overnight. The sample was lyophilized and dissolved in 0.1% formic acid. The digested sample was analyzed on liquid chromatography electrospray ionization mass spectrometry (LC-ESI-MS) on a Shimadzu Nexera Ultra HPLC (Japan) coupled to a TripleTOF 5600 mass spectrometer (ABSCIEX, Canada) equipped with a duo electrospray ion source. 3 µl of each extract was injected onto a 2.1×100 mm Zorbax C18 1.8 µm column (Agilent) at 400 µl/min. Linear gradients of 1–40% solvent B over 25 min at 400 µL/min flow rate, followed by a steeper gradient from 40% to 80% solvent B in 15 min were used for peptide elution. Solvent B was held at 80% for 5 min for washing the column and returned to 1% solvent B for equilibration prior to the next sample injection. Solvent A consisted of 0.1% formic acid (aqueous) and solvent B contained 90% *v/v* acetonitrile and 10% *v/v* 0.1% formic acid (aqueous). The ion spray voltage was set to 5300V, declustering potential (DP) 100V, curtain gas flow 25, nebuliser gas 1 (GS1) 25, GS2 to 35, interface heater at 150°C and the turbo heater to 450°C. The mass spectrometer acquired 250 ms full scan TOF-MS data followed by 20 by 50 ms full scan product ion data in an Information Dependant Acquisition (IDA) mode. Full scan TOFMS data was acquired over the mass range 350–1800 and for product ion ms/ms 100–1800. Ions observed in the TOF-MS scan exceeding a threshold of 200 counts and a charge state of +2 to +5 were set to trigger the acquisition of product ion, MS/MS spectra of the resultant 20 most intense ions. The data were acquired and processed using Analyst TF 1.5.1 software (ABSCIEX, Canada).

The assignment of cross-linked peptides was performed using the xBobcat version of the program xQuest [16]. Search parameters included the fix modification of Cys alkylation (mass shift of 57.02 Da), mass accuracy of 50 ppm and BS3 mono-links with hydrolyzed end (mass shift of 156.08 Da) and BS3 cross-links (mass shift of 138.07 Da). The potential cross-links were verified by manually inspecting the fragment ions of cross-linked peptides in the ESI mass spectra. The reported cross-links (Table 1) were verified by the presence of y- and/or b-ions for each peptide of the cross-linked peptides.

### SEC analysis of AG22 and Rac1 interaction

Purified AG22 (1–422) and Rac1.GDP at a 1∶1 molar ratio were incubated overnight at 4°C in SEC buffer (25 mM Tris-HCl, pH 7.5, 100 mM NaCl, 5 mM MgCl_2_ and 2 mM β-ME) with 1 mM AlCl_3_ and 20 mM NaF. The mixture of two proteins in a volume of 250 µL was then applied to a Superdex S200 (10/300GL) column using SEC buffer with 20 mM NaF.

## Supporting Information

Figure S1 **GST-ARHGAP22 (1–422) (S16D/S408D) pull-down assay.** GST-ARHGAP22 (1–422) (S16D/S408D) was immobilised on glutathione-sepharose beads and incubated overnight at 4°C with purified human 14-3-3. This GST pull-down experiment did not detect binding of 14-3-3. The experiments shown are representative of three replicates. The beads were analysed by SDS-PAGE and Coomassie staining.
(TIF)

Figure S2 **SAXS titration data suggest a weak interaction between AG22 (1–422) S16D/S411D and 14-3-3_2_.** Small-angle scattering data for mixtures of AG22 (1–422) S16D/S411D (at 26.5 µM) and 14-3-3_2_ at a range of molar ratios. Data were fit as linear combinations of scattering profiles from 14-3-3_2_, AG22 (1–422) S16D/S411D and cross-linked complex, yielding an estimate of the amount of each component in solution (Table S2).
(TIF)

Figure S3 **Cross-linking of AG22 (1–422) wild type with 14-3-3.** AG22 (1–422) wildtype (WT) at 10 µM and 14-3-3 (30 µM) were incubated with 3 mM BS3 for 5 or 30 min at room temperature following the methods described in Materials and Methods. The mixtures before and after cross-linking were analyzed by SDS-PAGE and visualized by Coomassie Blue staining. The results show that AG22 (1–422) WT can be crosslinked with 14-3-3.
(TIF)

Figure S4 **SEC analysis of AG22 (1–145) S16D and 14-3-3.** **A.** SEC elution profile for cross-linked (black) and uncross-linked (gray) complexes. A mixture of the two proteins or the purified cross-linked complex (250 µL) was injected onto a Superdex S200 (10/300GL) column; 0.5 mL fractions were collected and absorption (mAU) at 280 nm was monitored. The cross-linked complex of AG22 (1–145) S16D and 14-3-3 eluted earlier than the uncross-linked complex, at a mass a little larger than 66 kDa (consistent with the calculated mass of the complex of 80 kDa). The mixture of the two proteins eluted as two peaks: peak1 elutes at a mass of less than 66 kDa, consistent with the mass of the 14-3-3 dimer (2×29.3 kDa), and peak 2 elutes at a mass of ∼15 kDa consistent with the mass of an AG22 (1–145) (S16D) monomer (19.2 kDa). **B.** Peak fractions from the SEC experiment were analysed by SDS-PAGE. A 15 µL aliquot of each fraction was loaded onto the gel, protein components separated by electrophoresis and then visualized by Coomassie Blue staining. Molecular weights (kDa) of markers are indicated on the left and the proteins on the right.
(TIF)

Figure S5 **Solution structures of 14-3-3_2_, AG22 (1–422) S16D/S411D and AG22 (1–422) S16D/S411D:14-3-3_2_ cross-linked complex, optimized against scattering data.** **A.** The modeled 14-3-3_2_ solution structure optimized against SAXS data. The two monomers of the dimer are shown in green and blue. The enlarged region highlights the difference between the SAXS model and a crystal structure of 14-3-3 (red, PDB ID: 2BQ0). The bottom-most red helix in the crystal structure forms part of the peptide binding groove; the position of this helix differs in the solution structure. This difference may indicate that this C-terminal region is flexible and that peptide-binding stabilizes the helix position. **B.** Model of the AG22 (1–422) S16D/S411D solution structure optimized against SAXS data, showing the PH domain in orange and RhoGAP domain in red. The Cα atoms of the two phospho-mimic residues S16D and S411D are shown as magenta spheres. The distance between the geometric centres of the PH and RhoGAP domains is indicated **C.** The different classes of models obtained from rigid body optimization of the AG22 (1–422) S16D/S411D:14-3-3_2_ cross-linked complex against X-ray scattering data. For clarity the N-terminal, C-terminal and linker regions of AG22 are not shown. The PH domain of AG22 (orange) interacts with 14-3-3_2_ (gray) in a similar position in all models. The location of the RhoGAP domain differs in each model, where the RhoGAP position corresponding to the lowest *χ* ^2^ is shown in red (best model); positions of RhoGAP domains from other models are shown in magenta, pink and salmon (clockwise from the best model). The distance between the geometric centres of the PH and RhoGAP domains is indicated for each model.
(TIF)

Figure S6 **SEC analysis of AG22 (1–422) and Rac1.GDP.** **A.** SEC Elution profile. Purified AG22 (1–422) and Rac1.GDP at a 1∶1 molar ratio were incubated overnight at 4°C in the presence of 2 mM AlCl_3_ and 20 mM NaF. The mixture of the proteins was then injected onto a Superdex S200 (10/300GL) column; 0.5 mL fractions were collected and absorption (mAU) at 280 nm was monitored. The two proteins eluted as separate peaks, indicating that they did not form a stable complex under the conditions we used. Peak1 corresponded to AG22 (1–422) and peak 2 to Rac1. **B.** The peak fractions from SEC were analysed by SDS-PAGE. A 15 µL aliquot of each fraction was loaded onto the gel, components separated by electrophoresis and visualized by Coomassie Blue staining. Molecular weights (kDa) of markers are indicated on the left and the proteins are indicated on the right. A sample of the protein mixture injected onto the column is also shown.
(TIF)

Figure S7 **Cross-linking of Rac1 and AG22 constructs.** Each truncation construct of AG22 was mixed with Rac1 at equimolar concentrations and incubated with BS3 for 10 or 30 mins at room temperature, following the procedures described in the Materials and Methods. Samples were analyzed by SDS-PAGE and visualized with Coomassie Blue Staining. No cross-linked products were detected.
(TIF)

Table S1 **Structural parameters derived from scattering data and crystal structure data.**
(DOCX)

Table S2 **Concentrations and equilibrium constant for each point in the SAXS titration series.**
(DOCX)

## References

[1] Vetter IR, Wittinghofer A (2001) The guanine nucleotide-binding switch in three dimensions. Science 294: 1299–1304. 11701921 10.1126/science.1062023

[2] Iden S, Collard JG (2008) Crosstalk between small GTPases and polarity proteins in cell polarization. Nat Rev Mol Cell Biol 9: 846. 18946474 10.1038/nrm2521

[3] Bos JL, Rehmann H, Wittinghofer A (2007) GEFs and GAPs: Critical elements in the control of small G proteins. Cell 129: 865–877. 17540168 10.1016/j.cell.2007.05.018

[4] Moon SY, Zheng Y (2003) Rho GTPase-activating proteins in cell regulation. Trends Cell Biol 13: 13–22. 12480336 10.1016/s0962-8924(02)00004-1

[5] Sanz-Moreno V, Gadea G, Ahn J, Paterson H, Marra P, et al (2008) Rac activation and inactivation control plasticity of tumor cell movement. Cell 135: 510–523. 18984162 10.1016/j.cell.2008.09.043

[6] Rowland AF, Larance ML, Hughes WE, James DE (2011) Identification of RhoGAP22 as an Akt-dependent regulator of cell motility in response to insulin. Mol Cell Biol 31: 4789–4800. 21969604 10.1128/MCB.05583-11 PMC3232915

[7] Dougherty MK, Morrison DK (2004) Unlocking the code of 14-3-3. J Cell Sci 117: 1875–1884. 15090593 10.1242/jcs.01171

[8] Yaffe MB, Elia AEH (2001) Phosphoserine/threonine-binding domains. Curr Opin Cell Biol 13: 131–138. 11248545 10.1016/s0955-0674(00)00189-7

[9] Yang X, Lee WH, Sobott F, Papagrigoriou E, Robinson CV, et al (2006) Structural basis for protein–protein interactions in the 14-3-3 protein family. Proc Natl Acad Sci USA 103: 17237–17242. 17085597 10.1073/pnas.0605779103 PMC1859916

[10] Ottmann C, Yasmin L, Weyand M, Veesenmeyer JL, Diaz MH, et al (2007) Phosphorylation-independent interaction between 14-3-3 and exoenzyme S: from structure to pathogenesis. EMBO J 26: 902–913. 17235285 10.1038/sj.emboj.7601530 PMC1794388

[11] Jin JF, Smith D, Stark C, Wells CD, Fawcett JP, et al (2004) Proteomic, functional, and domain-based analysis of in vivo 14-3-3 binding proteins involved in cytoskeletal regulation and cellular organization. Curr Biol 14: 1436–1450. 15324660 10.1016/j.cub.2004.07.051

[12] Larance M, Rowland AF, Hoehn KL, Humphreys DT, Preiss T, et al (2010) Global phosphoproteomics identifies a major role for AKT and 14-3-3 in regulating EDC3. Mol Cell Proteomics 9: 682–694. 20051463 10.1074/mcp.M900435-MCP200 PMC2860230

[13] Obenauer JC, Cantley LC, Yaffe MB (2003) Scansite 2.0: proteome-wide prediction of cell signaling interactions using short sequence motifs. Nucleic Acids Res 31: 3635–3641. 12824383 10.1093/nar/gkg584 PMC168990

[14] Yaffe MB, Leparc GG, Lai J, Obata T, Volinia S, et al (2001) A motif-based profile scanning approach for genome-wide prediction of signaling pathways. Nat Biotechnol 19: 348–353. 11283593 10.1038/86737

[15] Perkins JR, Diboun I, Dessailly BH, Lees JG, Orengo C (2010) Transient protein-protein interactions: structural, functional, and network properties. Structure 18: 1233–1243. 20947012 10.1016/j.str.2010.08.007

[16] Rinner O, Seebacher J, Walzthoeni T, Mueller LN, Beck M, et al (2008) Identification of cross- linked peptides from large sequence databases. Nat Methods 5: 315–318. 18327264 10.1038/nmeth.1192 PMC2719781

[17] Petoukhov MV, Svergun DI (2005) Global rigid body modeling of macromolecular complexes against small-angle scattering data. Biophys J 89: 1237–1250. 15923225 10.1529/biophysj.105.064154 PMC1366608

[18] Ménétrey J, Perderiset M, Cicolari J, Dubois T, Elkhatib N, et al (2007) Structural basis for ARF1-mediated recruitment of ARHGAP21 to Golgi membranes. EMBO J 26: 1953–1962. 17347647 10.1038/sj.emboj.7601634 PMC1847662

[19] Würtele M, Wolf E, Pederson KJ, Buchwald G, Ahmadian MR, et al (2001) How the Pseudomonas aeruginosa ExoS toxin downregulates Rac. Nat Struct Biol 8: 23–26. 11135665 10.1038/83007

[20] Aitken A (2006) 14-3-3 proteins: a historic overview. Semin Cancer Biol 16: 162–172. 16678438 10.1016/j.semcancer.2006.03.005

[21] Obsil T, Ghirlando R, Klein DC, Ganguly S, Dyda F (2001) Crystal structure of the 14-3-3zeta:serotonin N-acetyltransferase complex. A role for scaffolding in enzyme regulation. Cell 105: 257–267. 11336675 10.1016/s0092-8674(01)00316-6

[22] Gardino AK, Smerdon SJ, Yaffe MB (2006) Structural determinants of 14-3-3 binding specificities and regulation of subcellular localization of 14-3-3-ligand complexes: A comparison of the X-ray crystal structures of all human 14-3-3 isoforms. Semin Cancer Biol 16: 173–182. 16678437 10.1016/j.semcancer.2006.03.007

[23] Nishi H, Hashimoto K, Panchenko AR (2011) Phosphorylation in protein-protein binding: effect on stability and function. Structure 19: 1807–1815. 22153503 10.1016/j.str.2011.09.021 PMC3240861

[24] Geraghty KM, Chen S, Harthill JE, Ibrahim AF, Toth R, et al (2007) Regulation of multisite phosphorylation and 14-3-3 binding of AS160 in response to IGF-1, EGF, PMA and AICAR. Biochem J 407: 231–241. 17617058 10.1042/BJ20070649 PMC2049023

[25] Zhai J, Lin H, Shamim M, Schlaepfer WW, Cañete-Soler R (2001) Identification of a novel interaction of 14-3-3 with p190RhoGEF. J Biol Chem 276: 41318–41324. 11533041 10.1074/jbc.M107709200

[26] Ohta Y, Hartwig JH, Stossel TP (2006) FilGAP, a Rho- and ROCK-regulated GAP for Rac binds filamin A to control actin remodelling. Nat Cell Biol 8: 803–818. 16862148 10.1038/ncb1437

[27] Stols L, Gu M, Dieckman L, Raffen R, Collart FR, et al (2002) A new vector for high-throughput, ligation-independent cloning encoding a tobacco etch virus protease cleavage site. Protein expression and purification 25: 8–15. 12071693 10.1006/prep.2001.1603

[28] Studier FW (2005) Protein production by auto-induction in high density shaking cultures. Protein Expr Purif 41: 207–234. 15915565 10.1016/j.pep.2005.01.016

[29] Cookson DJ (2007). SAXS15ID - Software for acquiring, processing and viewing SAXS/WAXS image data at ChemMatCARS.

[30] Orthaber D, Bergmann A, Glatter O (2000) SAXS experiments on absolute scale with Kratky systems using water as a secondary standard. J Appl Crystallogr 33: 218–225.

[31] Svergun DI (1992) Determination of the regularization parameter in indirect-transform methods using perceptual criteria. J Appl Crystallogr 25: 495–503.

[32] Zhang Y (2008) I-TASSER server for protein 3D structure prediction. BMC Bioinformatics 9: 40–47. 18215316 10.1186/1471-2105-9-40 PMC2245901

[33] DeLano WL (2002) The PyMol molecular graphics system (San Carlos, CA: DeLano Scientific LLC)

