# Supplementary figures and images for: The Weak Complex between RhoGAP Protein ARHGAP22 and Signal Regulatory Protein 14-3-3 Has 1∶2 Stoichiometry and a Single Peptide Binding Mode
Source: PLoS One. 2012 Aug 28;7(8):e41731. doi: 10.1371/journal.pone.0041731 (PMC3429473; doi:10.1371/journal.pone.0041731)

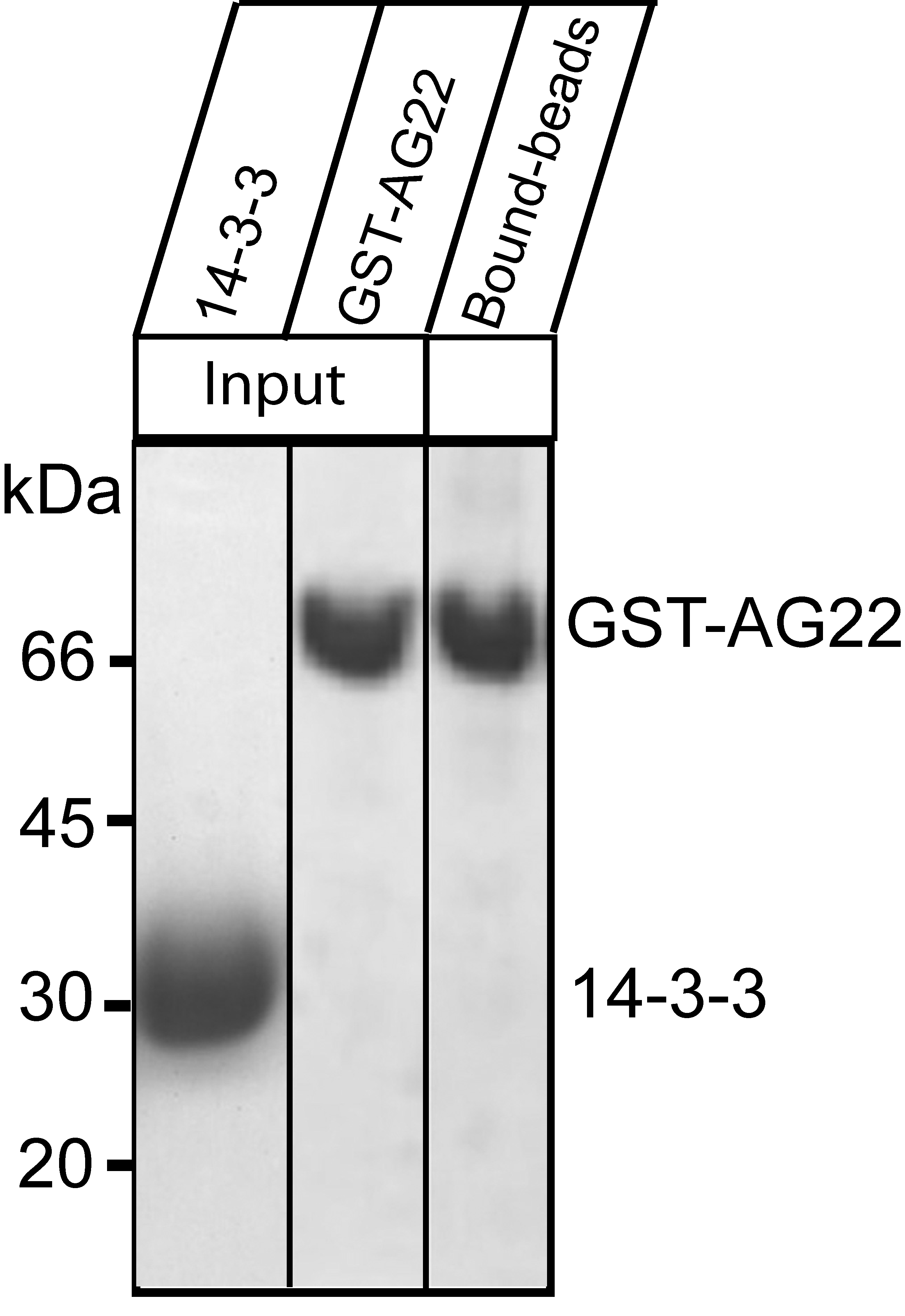

Supplement: Figure S1 — GST-ARHGAP22 (1–422) (S16D/S408D) pull-down assay. GST-ARHGAP22 (1–422) (S16D/S408D) was immobilised on glutathione-sepharose beads and incubated overnight at 4°C with purified human 14-3-3. This GST pull-down experiment did not detect binding of 14-3-3. The experiments shown are representative of three replicates. The beads were analysed by SDS-PAGE and Coomassie staining. (TIF) [file pone.0041731.s001.tif]

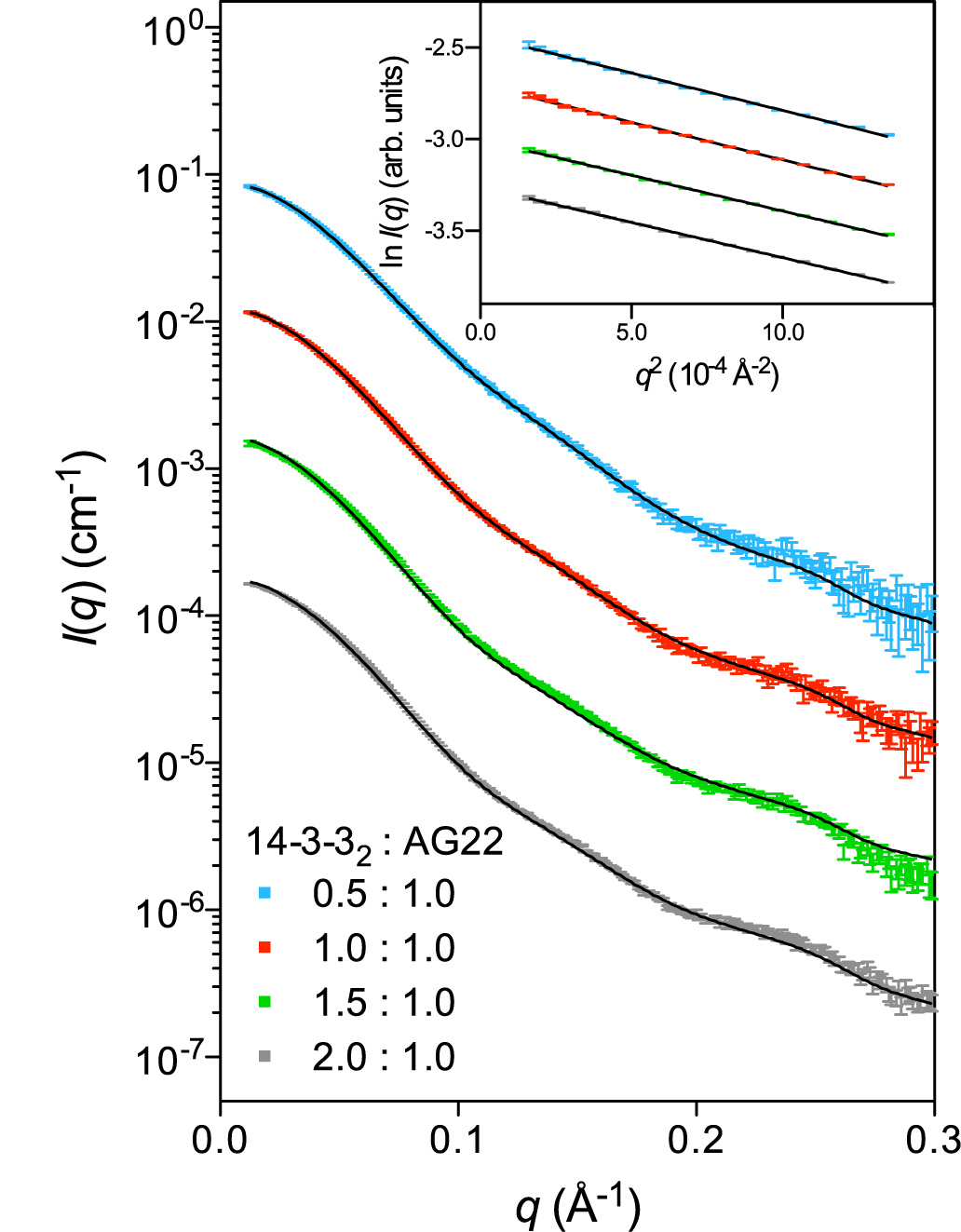

Supplement: Figure S2 — SAXS titration data suggest a weak interaction between AG22 (1–422) S16D/S411D and 14-3-32. Small-angle scattering data for mixtures of AG22 (1–422) S16D/S411D (at 26.5 µM) and 14-3-32 at a range of molar ratios. Data were fit as linear combinations of scattering profiles from 14-3-32, AG22 (1–422) S16D/S411D and cross-linked complex, yielding an estimate of the amount of each component in solution (Table S2). (TIF) [file pone.0041731.s002.tif]

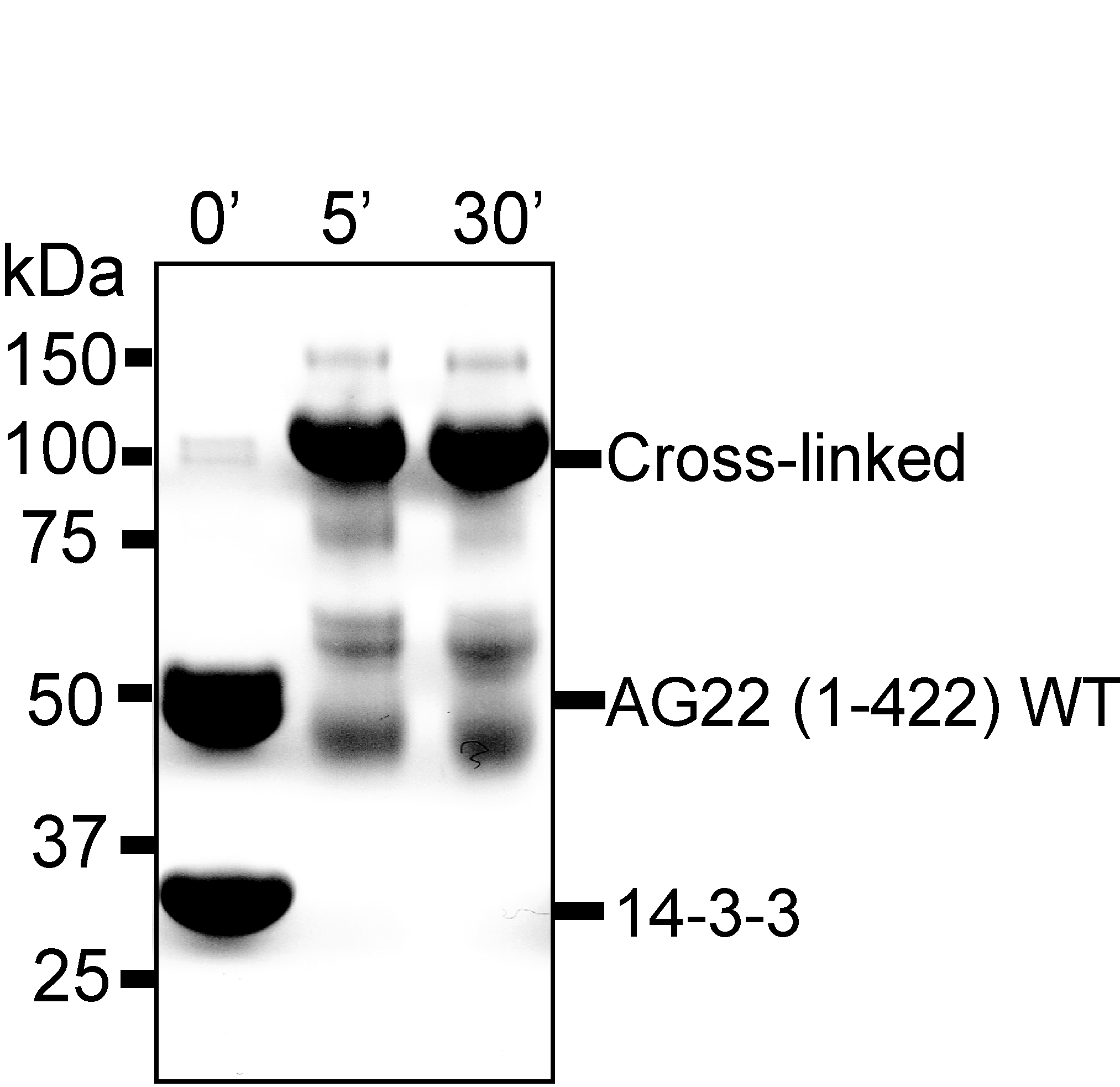

Supplement: Figure S3 — Cross-linking of AG22 (1–422) wild type with 14-3-3. AG22 (1–422) wildtype (WT) at 10 µM and 14-3-3 (30 µM) were incubated with 3 mM BS3 for 5 or 30 min at room temperature following the methods described in Materials and Methods. The mixtures before and after cross-linking were analyzed by SDS-PAGE and visualized by Coomassie Blue staining. The results show that AG22 (1–422) WT can be crosslinked with 14-3-3. (TIF) [file pone.0041731.s003.tif]

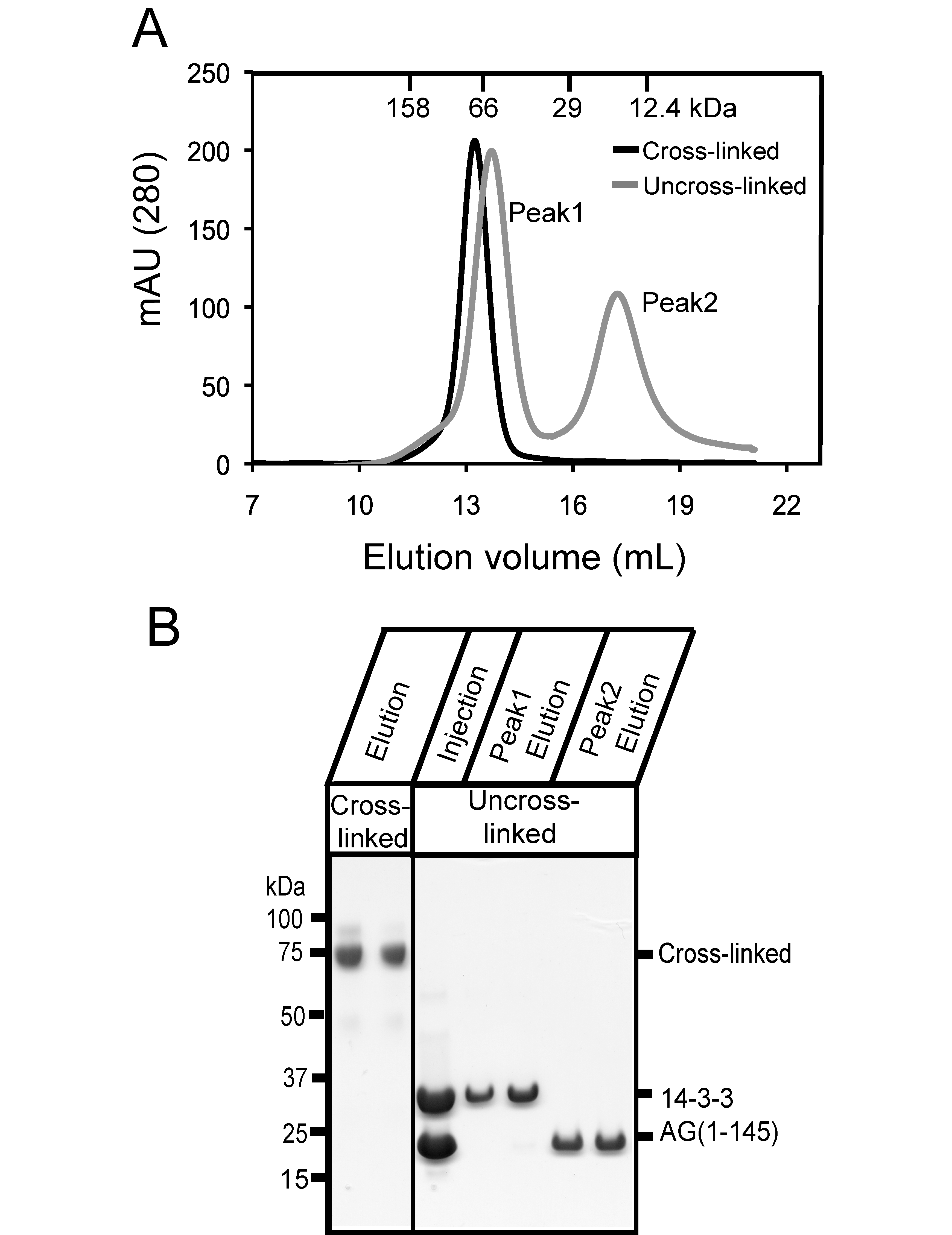

Supplement: Figure S4 — SEC analysis of AG22 (1–145) S16D and 14-3-3. A. SEC elution profile for cross-linked (black) and uncross-linked (gray) complexes. A mixture of the two proteins or the purified cross-linked complex (250 µL) was injected onto a Superdex S200 (10/300GL) column; 0.5 mL fractions were collected and absorption (mAU) at 280 nm was monitored. The cross-linked complex of AG22 (1–145) S16D and 14-3-3 eluted earlier than the uncross-linked complex, at a mass a little larger than 66 kDa (consistent with the calculated mass of the complex of 80 kDa). The mixture of the two proteins eluted as two peaks: peak1 elutes at a mass of less than 66 kDa, consistent with the mass of the 14-3-3 dimer (2×29.3 kDa), and peak 2 elutes at a mass of ∼15 kDa consistent with the mass of an AG22 (1–145) (S16D) monomer (19.2 kDa). B. Peak fractions from the SEC experiment were analysed by SDS-PAGE. A 15 µL aliquot of each fraction was loaded onto the gel, protein components separated by electrophoresis and then visualized by Coomassie Blue staining. Molecular weights (kDa) of markers are indicated on the left and the proteins on the right. (TIF) [file pone.0041731.s004.tif]

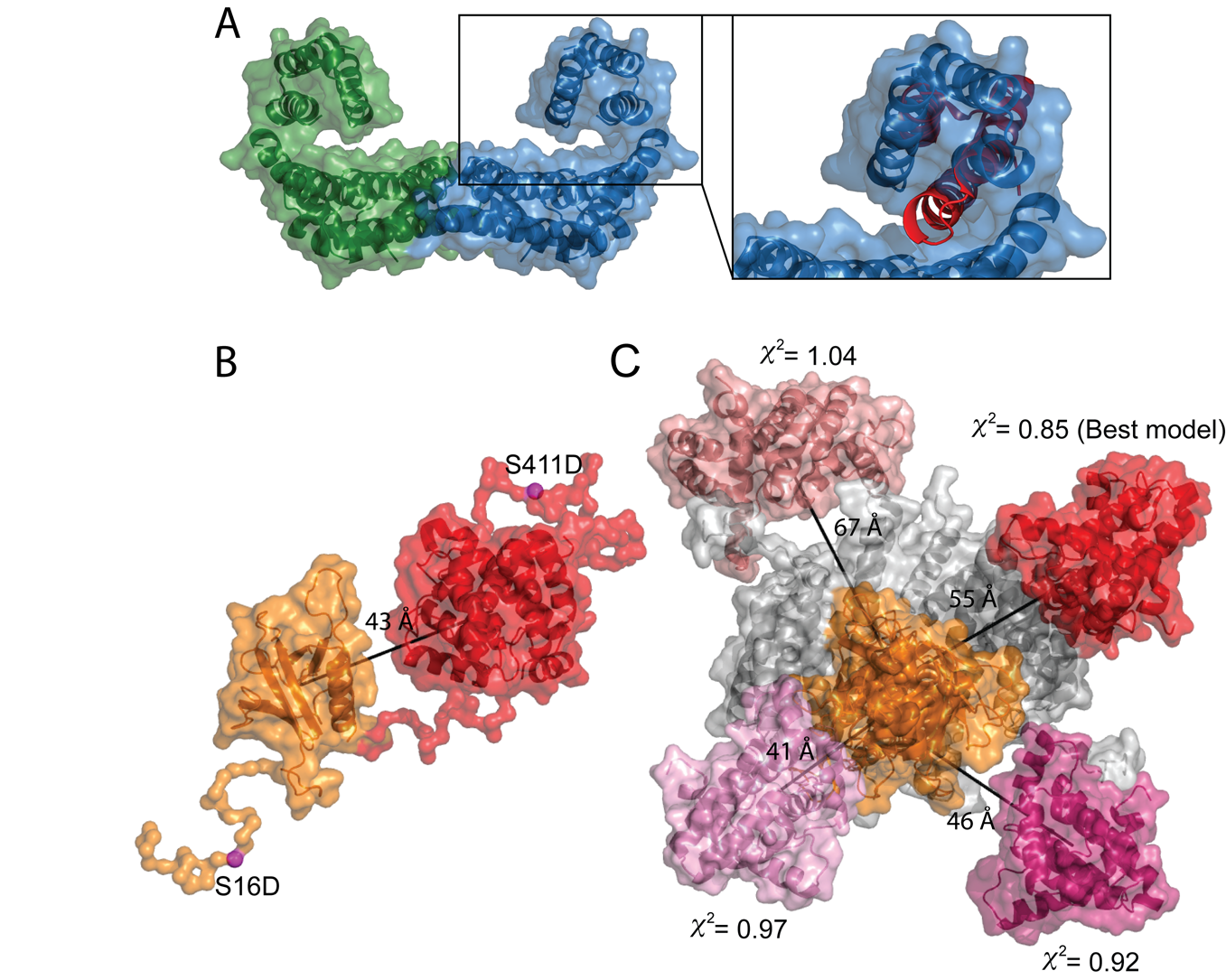

Supplement: Figure S5 — Solution structures of 14-3-32, AG22 (1–422) S16D/S411D and AG22 (1–422) S16D/S411D:14-3-32 cross-linked complex, optimized against scattering data. A. The modeled 14-3-32 solution structure optimized against SAXS data. The two monomers of the dimer are shown in green and blue. The enlarged region highlights the difference between the SAXS model and a crystal structure of 14-3-3 (red, PDB ID: 2BQ0). The bottom-most red helix in the crystal structure forms part of the peptide binding groove; the position of this helix differs in the solution structure. This difference may indicate that this C-terminal region is flexible and that peptide-binding stabilizes the helix position. B. Model of the AG22 (1–422) S16D/S411D solution structure optimized against SAXS data, showing the PH domain in orange and RhoGAP domain in red. The Cα atoms of the two phospho-mimic residues S16D and S411D are shown as magenta spheres. The distance between the geometric centres of the PH and RhoGAP domains is indicated C. The different classes of models obtained from rigid body optimization of the AG22 (1–422) S16D/S411D:14-3-32 cross-linked complex against X-ray scattering data. For clarity the N-terminal, C-terminal and linker regions of AG22 are not shown. The PH domain of AG22 (orange) interacts with 14-3-32 (gray) in a similar position in all models. The location of the RhoGAP domain differs in each model, where the RhoGAP position corresponding to the lowest χ 2 is shown in red (best model); positions of RhoGAP domains from other models are shown in magenta, pink and salmon (clockwise from the best model). The distance between the geometric centres of the PH and RhoGAP domains is indicated for each model. (TIF) [file pone.0041731.s005.tif]

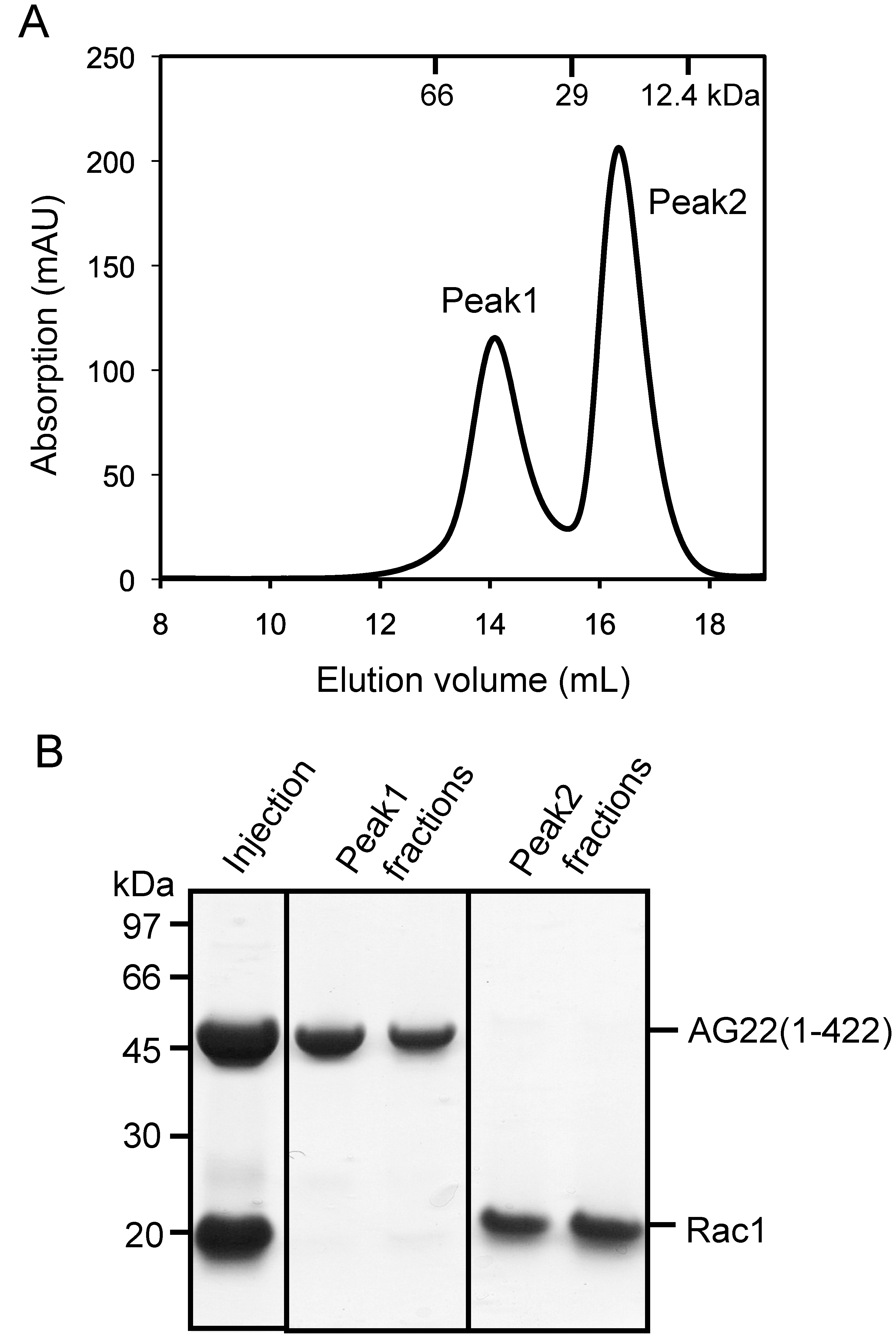

Supplement: Figure S6 — SEC analysis of AG22 (1–422) and Rac1.GDP. A. SEC Elution profile. Purified AG22 (1–422) and Rac1.GDP at a 1∶1 molar ratio were incubated overnight at 4°C in the presence of 2 mM AlCl3 and 20 mM NaF. The mixture of the proteins was then injected onto a Superdex S200 (10/300GL) column; 0.5 mL fractions were collected and absorption (mAU) at 280 nm was monitored. The two proteins eluted as separate peaks, indicating that they did not form a stable complex under the conditions we used. Peak1 corresponded to AG22 (1–422) and peak 2 to Rac1. B. The peak fractions from SEC were analysed by SDS-PAGE. A 15 µL aliquot of each fraction was loaded onto the gel, components separated by electrophoresis and visualized by Coomassie Blue staining. Molecular weights (kDa) of markers are indicated on the left and the proteins are indicated on the right. A sample of the protein mixture injected onto the column is also shown. (TIF) [file pone.0041731.s006.tif]

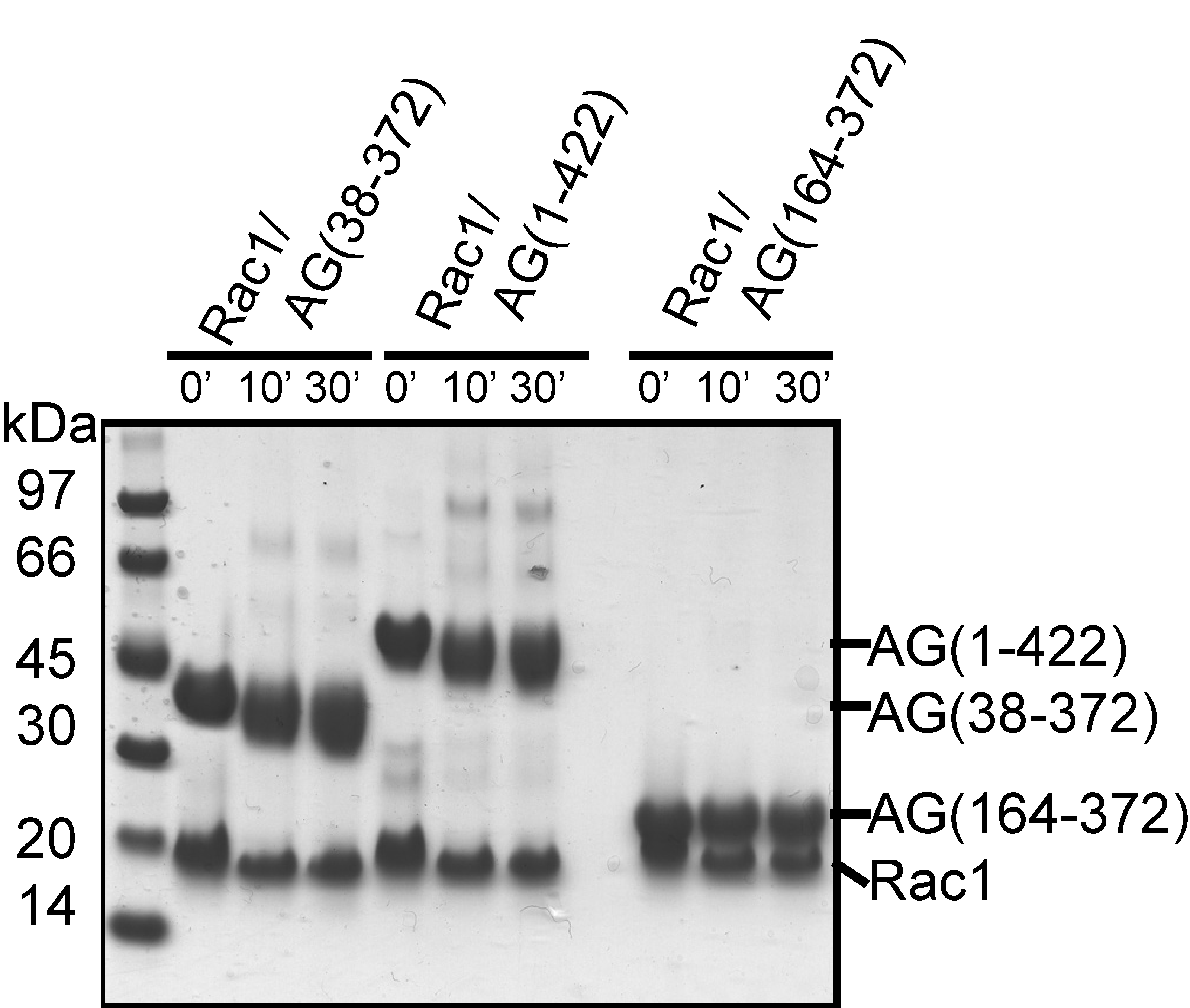

Supplement: Figure S7 — Cross-linking of Rac1 and AG22 constructs. Each truncation construct of AG22 was mixed with Rac1 at equimolar concentrations and incubated with BS3 for 10 or 30 mins at room temperature, following the procedures described in the Materials and Methods. Samples were analyzed by SDS-PAGE and visualized with Coomassie Blue Staining. No cross-linked products were detected. (TIF) [file pone.0041731.s007.tif]
