# Supplementary material for: The Weak Complex between RhoGAP Protein ARHGAP22 and Signal Regulatory Protein 14-3-3 Has 1∶2 Stoichiometry and a Single Peptide Binding Mode
Source: PLoS One. 2012 Aug 28;7(8):e41731. doi: 10.1371/journal.pone.0041731 (PMC3429473; doi:10.1371/journal.pone.0041731)
Supplement: Table S1 — Structural parameters derived from scattering data and crystal structure data. (DOCX) [file pone.0041731.s008.docx]

|  | Concn.^§^  (mg/mL) | *R*_g_  (Å) | *I*(0)  (cm^-1^) | *D*_max_^¶^  (Å) | Mass^#^  (kDa) |
| --- | --- | --- | --- | --- | --- |
| 14-3-3 | 1.50* | 30.3±0.2 | 0.0588±0.0001 | 100 | 55 |
|  | 2.80 | 30.2±0.1 | 0.1077±0.0002 | 100 | 54 |
|  | 5.80 | 29.8±0.1 | 0.2193±0.0003 | 100 | 53 |
|  | Crystal structure | 30.7 | – | 95 | – |
|  |  |  |  |  |  |
| AG22 (1-422) | 1.25* | 31.1±0.2 | 0.0406±0.0002 | 120 | 45 |
| S16D/S411D | 2.60 | 31.4±0.2 | 0.0858±0.0002 | 120 | 46 |
|  |  |  |  |  |  |
| Cross-linked | 0.15 | 37.8±1.5 | 0.0110±0.0004 | 145 | 102 |
| complex | 0.35* | 37.8±0.6 | 0.0265±0.0003 | 145 | 105 |
| **Titration series:** |  |  |  |  |  |
| 1:1 | 0.00 : 1.25 | 34.3±0.2 | 0.0865±0.0003 | 145 | – |
| 2:1 | 0.80 : 1.25 | 34.4±0.2 | 0.1212±0.0004 | 145 | – |
| 3:1 | 1.60 : 1.25 | 34.0±0.1 | 0.1575±0.0004 | 145 | – |
| 4:1 | 3.20 : 1.25 | 33.8±0.1 | 0.1726±0.0004 | 145 | – |

Structural parameters from scattering data were determined using GNOM [[1](#_ENREF_1)]. Structural parameters from the 14-3-3 crystal structure were determined using CRYSOL [[2](#_ENREF_2)]. The crystal structure used was PDB ID: 2BQ0 [[3](#_ENREF_3)]. ^§^Concentrations were determined by absorption at *λ* = 280 nm; ^¶^The uncertainty in *D*_max_ is approximately ± 5%; ^#^The mass of the particle is calculated as described elsewhere [[4](#_ENREF_4)] from the concentration, *I*(0) and contrast and partial specific volume calculated using MULCh [[5](#_ENREF_5)]. The expected masses are: 14-3-3_2_, 58.6 kDa; AG22 (1-422) S16D/S411D, 47.2 kDa; cross-linked complex, 105.8 kDa. *Indicates data used for modelling.

**REFERENCES**

1. Svergun DI (1992) Determination of the regularization parameter in indirect-transform methods using perceptual criteria. J Appl Crystallogr 25: 495-503.

2. Svergun D, Barberato C, Koch MHJ (1995) CRYSOL - A program to evaluate x-ray solution scattering of biological macromolecules from atomic coordinates. J Appl Crystallogr 28: 768-773.

3. Yang X, Lee WH, Sobott F, Papagrigoriou E, Robinson CV, et al. (2006) Structural basis for protein–protein interactions in the 14-3-3 protein family. Proc Natl Acad Sci USA 103: 17237–17242.

4. Orthaber D, Bergmann, A. and Glatter, O. (2000) SAXS experiments on absolute scale with Kratky systems using water as a secondary standard. J Appl Crystallogr 33: 218-225.

5. Whitten AE, Cai, S.Z. and Trewhella, J. (2008) MULCh: modules for the analysis of small-angle neutron contrast variation data from biomolecular assemblies. . J Appl Crystallogr 41: 222-226.
