# Supplementary material for: The Weak Complex between RhoGAP Protein ARHGAP22 and Signal Regulatory Protein 14-3-3 Has 1∶2 Stoichiometry and a Single Peptide Binding Mode
Source: PLoS One. 2012 Aug 28;7(8):e41731. doi: 10.1371/journal.pone.0041731 (PMC3429473; doi:10.1371/journal.pone.0041731)
Supplement: Table S2 — Concentrations and equilibrium constant for each point in the SAXS titration series. (DOCX) [file pone.0041731.s009.docx]

| Titration Point | [14-3-3_2_]  (μM) | [AG22]  (μM) | [AG22:14-3-3_2_]  (μM) | *K*_d_  (μM) |
| --- | --- | --- | --- | --- |
| 0.5:1.0 | 7.5 | 20.8 | 5.7 | 27.4 |
| 1.0:1.0 | 17.0 | 17.0 | 9.5 | 30.6 |
| 1.5:1.0 | 25.5 | 12.2 | 14.3 | 20.9 |
| 2.0:1.0 | 39.1 | 12.6 | 13.9 | 34.2 |
| Average |  |  |  | 28±6* |

Equilibrium concentrations of 14-3-3_2_, AG22 (AG22 (1-422) S16D/S411D) and AG22:14-3-3_2_ (the AG22 (1-422) S16D/S411D:14-3-3_2_ complex) and the dissociation constant (*K*_d_), estimated from small-angle X-ray scattering titration experiments, were calculated for each titration point as described in Experimental Procedures.*The quoted uncertainty for the average *K*_d_ is the standard deviation.
